# Constructing, Perceiving, and Maintaining Scenes: Hippocampal Activity and Connectivity

**DOI:** 10.1093/cercor/bhu266

**Published:** 2014-11-18

**Authors:** Peter Zeidman, Sinéad L. Mullally, Eleanor A. Maguire

**Affiliations:** Wellcome Trust Centre for Neuroimaging, Institute of Neurology, University College London, London WC1N 3BG, UK

**Keywords:** episodic memory, fMRI, hippocampus, perception, scene construction, scenes

## Abstract

In recent years, evidence has accumulated to suggest the hippocampus plays a role beyond memory. A strong hippocampal response to scenes has been noted, and patients with bilateral hippocampal damage cannot vividly recall scenes from their past or construct scenes in their imagination. There is debate about whether the hippocampus is involved in the online processing of scenes independent of memory. Here, we investigated the hippocampal response to visually perceiving scenes, constructing scenes in the imagination, and maintaining scenes in working memory. We found extensive hippocampal activation for perceiving scenes, and a circumscribed area of anterior medial hippocampus common to perception and construction. There was significantly less hippocampal activity for maintaining scenes in working memory. We also explored the functional connectivity of the anterior medial hippocampus and found significantly stronger connectivity with a distributed set of brain areas during scene construction compared with scene perception. These results increase our knowledge of the hippocampus by identifying a subregion commonly engaged by scenes, whether perceived or constructed, by separating scene construction from working memory, and by revealing the functional network underlying scene construction, offering new insights into why patients with hippocampal lesions cannot construct scenes.

## Introduction

The neuroimaging literature is replete with studies showing that posterior parahippocampal cortex (PHC) and retrosplenial cortex (RSC) are more engaged by scenes than other types of stimuli (Epstein and Kanwisher 1998; Epstein 2008; Ranganath and Ritchey 2012). While PHC and RSC involvement in processing scene-related information is not in doubt, in recent years the hippocampus has also been linked with scenes.

Patients with bilateral hippocampal damage and amnesia are not only impaired at recalling events from their past, but also at imagining events in their personal future. Klein et al. (2002) described patient D.B., who was amnesic and could not imagine his personal past or future, but had preserved semantic knowledge and could use it to reason about general (nonpersonal) past and future events. Andelman et al. (2010) reported the case of patient M.C. who, following an epileptic episode, experienced bilateral hippocampal damage and similarly could not recall years of her past or imagine her personal future. To investigate the shared process which may underlie recalling past and imagining future scenes, Hassabis, Kumaran, and Vann et al. (2007) asked a group of patients with bilateral hippocampal damage and amnesia to construct atemporal scenes (i.e., not set in the past or the future) in their imagination. The patients were impaired at constructing scenes relative to matched control subjects (replicated by Mullally, Intraub, and Maguire 2012) with a specific deficit noted in the spatial coherence of their imagined experiences. This suggested that the hippocampus may perform a common function for the construction of scenes in the imagination, regardless of whether they are memories from one's past, atemporal fictitious events, or events set in one's personal future (Hassabis and Maguire 2007, 2009; Maguire and Mullally 2013). Neuroimaging studies have since confirmed hippocampal engagement during the construction of static scenes (Hassabis, Kumaran, and Maguire 2007) and the construction of future episodes (Addis, Wong et al. 2007) in healthy controls, and have demonstrated a network of co-activated regions including hippocampus, PHC, RSC, and medial prefrontal cortex.

As well as the active construction of scenes, the role of the hippocampus in perceiving scenes has stimulated significant debate. A series of studies have demonstrated that patients with hippocampal lesions are impaired at discriminating scenes but not other forms of visual stimuli (Lee, Buckley et al. 2005; Lee, Bussey et al. 2005), as well as identifying a hippocampal response to scene discrimination in neurologically healthy participants (Lee et al. 2008; Barense et al. 2010). Those authors suggest the hippocampus may provide an allocentric or viewpoint-independent representation of the scenes which facilitates discrimination, performing pattern separation to distinguish highly similar spatial configurations (Lee et al. 2012). Perceptual and memory deficits following medial temporal lobe (MTL) damage occur, they suggest, because complex conjunctions of spatial information in hippocampus and object information in perirhinal cortex are lost (Graham et al. 2010). A separate line of evidence has also implicated the hippocampus in scene perception. Patients with hippocampal lesions show attenuated boundary extension (BE) (Intraub and Richardson 1989)—the automatic process of extrapolating beyond the view in scenes (Mullally, Intraub, and Maguire 2012). Chadwick et al. (2013) found the hippocampus to be engaged when healthy controls viewed simple scenes and BE occurred. Thus, the hippocampus may be involved in the perception of scenes—either when comprehending the current scene in view, or predicting beyond the edges of the scene.

A link between perception and imagination was highlighted by Gaesser et al. (2011), who investigated the level of detail included in narrative descriptions of scene photographs, imagined events, and autobiographical memories, in healthy young and older adults. Following an established protocol (Levine et al. 2002; Addis et al. 2008), they divided narratives into episodic “internal” details (e.g., who, what, where, when) and semantic “external” details. They found that the number of episodic details in the descriptions of photographs, which declined with age, predicted the number of episodic details for imagined events and for autobiographical memories, suggesting a common process involved with scene perception, imagination, and autobiographical memory.

There has been significant opposition to studies demonstrating hippocampal involvement in scene processing, with other reports showing that patients with hippocampal lesions are not impaired at discrimination tasks (Shrager et al. 2006; Kim et al. 2011). It has been concluded from these studies that previous findings of deficits can be explained by patients' inability to learn across trials, long-term memory (LTM) encoding difficulties, and unidentified lesions beyond the hippocampus (Shrager et al. 2006; Suzuki 2009; Kim et al. 2011; Knutson et al. 2012). There is further evidence that patients with hippocampal lesions appear to have intact scene perception. Race et al. (2011) used a similar protocol to Gaesser et al. (2011), although with simple cartoon stimuli rather than scene photographs, in order to test the scene perception of 8 amnesic patients with hippocampal damage. They found that while the patients had impoverished descriptions of personal past and future events, their ability to describe narratives for cartoon pictures placed in front of them was matched to controls. This suggested that the hippocampus may not be required for processing the scene currently in view. Mullally, Intraub, and Maguire (2012) similarly tested 7 patients with focal bilateral hippocampal damage and amnesia on their ability to describe a scene photograph. They found that participants could accurately describe a photograph, and they could reason about what objects may come into view if the participant imagined that the standpoint of the picture was moved backward by a few paces. However, in contrast to healthy controls, the patients omitted references to the spatial arrangement of objects beyond the edges of a picture, suggesting that the hippocampus may only be required for processing the scene beyond the current view. Although one study found that hippocampal patients were impaired in describing real-life and pictorial scenes (Zeman et al. 2013), this was based on patients with mixed etiology, no structural detail on their lesions was provided and this could not be replicated in a subsequent study by Race et al. (2013). In summary, there is a lack of consensus in the literature over whether the hippocampus is involved in scene perception, and this is complicated by potential confounds such as memory encoding.

On the basis that scene construction engages the hippocampus during imagination (Hassabis, Kumaran, Vann, et al. 2007, Hassabis, Kumaran, and Maguire 2007), we hypothesized that, in neurologically healthy participants, simply perceiving scenes (without a comparison or discrimination task) may engage the hippocampus, reflecting the creation of an internal model of the scene. Under this hypothesis, differences in findings across studies on scene perception would be due to whether or not participants constructed a spatially coherent model of the scene on each trial of the experiment. This idea accords with recent findings that the hippocampus is engaged by discriminating scenes based on their global configuration, or “strength-based perception,” but not when discriminations are based on differences in local visual features, or “state-based perception” (Aly and Yonelinas 2012; Aly et al. 2013). Similarly, Hartley et al. (2007) proposed that the hippocampus would be engaged “when a flexible or allocentric representation of spatial layout is required.” In this study, for the first time, we set out to directly compare scene perception and scene construction to test whether there is evidence that both functions share a neural substrate involving, in particular, the hippocampus.

We also took this opportunity to investigate a second aspect of scene construction. When someone constructs a scene in his or her imagination, in addition to creating the scene representation, he or she must also maintain it in working memory. While the established model of hippocampal function is that it is not involved in working memory (Cave and Squire 1992; Alvarez et al. 1994), this has been challenged in recent years (see Ranganath and Blumenfeld 2005 for review) and is a topic of active debate. For instance, Hannula et al. (2006) reported that patients with hippocampal lesions had impairments in maintaining spatial relations between items within scenes as well as face–scene associations, which they suggested showed a more general impairment in short-term relational memory. Several studies have shown an interaction between stimulus type (scene or non-scene) and working memory load (Stern et al. 2001; Hartley et al. 2007; Lee and Rudebeck 2010), and others have suggested a role for the hippocampus in maintaining object–location associations (Olson, Moore, et al. 2006; Olson, Page, et al. 2006). Cashdollar et al. (2009) used MEG to investigate scene maintenance in controls and patients with bilateral hippocampal sclerosis, and found increased synchronicity of occipital and temporal regions with hippocampal theta during maintenance of spatial (configural) information about the scene, suggesting the hippocampus may mediate the maintenance of scenes in working memory. In contrast, Schon et al. (2009) found no effect of working memory load for scenes during working memory maintenance. Jeneson and Squire (2012) suggested that it is a question of capacity—if a task requires more than can be held in the limited capacity of working memory, then LTM will be required, which engages the medial temporal lobes. However, they gave no consideration of how the content of the maintained material might modulate hippocampal activation. In reviewing the same literature, Yonelinas (2013) discussed experiments using scene stimuli which indicated a working memory deficit following hippocampal lesions, although this was viewed as part of a wider notion that the hippocampus supports “high-resolution bindings.” Given the apparent hippocampal preference for scenes already discussed, it seems crucial to consider the nature of the stimuli and not just the working memory load. Surprisingly, many fMRI studies on scene maintenance either have not examined or did not report data from the hippocampus (e.g., Johnson et al. 2007; Yi et al. 2008; Park et al. 2010), a limitation we aimed to address in the current study. We did not set out to characterize all aspects of working memory, rather we aimed to address a specific question—is working memory maintenance sufficient to explain the hippocampal response to scene construction? We hypothesized that scene maintenance would not be sufficient to explain activations associated with scene construction, and thus scene maintenance would result in significantly less activation of the hippocampus, if any.

The third aim of this study was to explore the functional connectivity of the hippocampus during scene construction and scene perception. Little is known about the connectivity of brain regions during scene processing, and this is especially true of the connectivity between cortex and hippocampus which has largely been overlooked by the recent trend of studies examining large-scale brain networks (Bullmore and Sporns 2009). Initial insights come from 2 recent studies. Chadwick et al. (2013) applied dynamic causal modeling and found that the hippocampus had a top-down influence on early visual areas during BE. Aly et al. (2013) used a psychophysiological interaction (PPI) analysis to demonstrate greater interaction between hippocampus and lingual gyrus with confidence judgments in a scene discrimination task. Taking our lead from these studies looking at task-related changes in connectivity, we hoped to gain better insights into the relationship between the hippocampus and other regions of the network engaged during scene construction (Hassabis, Kumaran, and Maguire 2007; Summerfield et al. 2010), including RSC, precuneus, PHC, and medial prefrontal cortex. While there is anatomical evidence for indirect anatomical pathways linking early visual areas to the hippocampus (Kravitz et al. 2011), there is limited evidence for the functional connections which would be employed during scene perception, and we were interested to test for similarities and differences in connectivity between scene perception and scene construction.

We hypothesized that constructing scenes would induce connectivity between the hippocampus and regions performing 2 functions. The first would be those which store elements of the scene which would be brought together into a coherent representation. For instance, semantic information would be provided by lateral temporal lobe regions, including anterior middle temporal gyrus and superior temporal sulcus which have been implicated in autobiographical memory (Svoboda et al. 2006) and imagining (Addis et al. 2009) and have been suggested to act as an amodal hub for linking together information of different modalities into unified concepts (Binder et al. 2009; Binney et al. 2010; Pobric et al. 2010). We also expected strong connectivity between hippocampus and perirhinal cortex/lateral occipital cortex to provide object information; however, our use of single-object baselines may preclude this being observed. The second category of regions we expected were in the visual processing hierarchy, in order to support vivid re-experiencing of the scenes. These may be primary occipital regions, following previous observations of re-activation of occipital cortex in imagination (Ganis et al. 2004; Slotnick et al. 2005), connectivity with lingual gyrus as shown using scene discrimination in Aly et al. (2013) and top-down influence of the hippocampus on early occipital cortex shown during BE (Chadwick et al. 2013). We also expected to see regions along the dorsal visual pathway including parietal and retrosplenial regions, which may facilitate the interface between allocentric hippocampal representations and egocentric visual stimuli (Burgess et al. 2001; Byrne et al. 2007; Auger et al. 2012).

A dearth of previous data makes predicting hippocampal connectivity during scene perception less certain. If early visual regions are driven in a top-down fashion by a hippocampal model when imagining scenes, as we hypothesize, then we would expect weaker connectivity with the hippocampus when they are driven by real visual stimuli. Even if scene perception does involve the creation of a hippocampus-dependent model of the scene, it is uncertain whether passive scene viewing would tax the hippocampus to as great an extent as constructing scenes in the imagination. Thus, how the strength of connections would compare between these conditions was an open question. For scene maintenance, if hippocampal engagement was found, we expected this to be with regions of the “dorsal attention network” (Gitelman et al. 1999; Corbetta and Shulman 2002; Ptak and Schnider 2010).

To summarize, there is an increasing appreciation that the mental simulation of scenes may be central to hippocampal function (Hassabis and Maguire 2007, 2009; Schacter et al. 2012; Maguire and Mullally 2013); however, there are gaps in our knowledge. In this fMRI study, we set out to characterize the hippocampal response to scenes by directly comparing scene perception, scene construction, and the maintenance of scenes in working memory. We addressed 3 questions. First, does scene perception engage the hippocampus? Second, when constructing (imagining) scenes, is the hippocampus involved with the initial construction of the scene, or with maintaining the scene representation in working memory, or with both? And third, we explored a related issue about which little is currently known, namely, what is the hippocampal functional connectivity with the rest of the brain during scene perception, scene construction, and scene maintenance?

## Materials and Methods

### Participants

There were 25 healthy, right-handed participants (10 males, mean age 26.16 years, SD 4.38). All had normal or corrected-to-normal vision, and gave informed written consent to participation in accordance with the University College London research ethics committee.

### Tasks and Procedure

Prior to scanning, participants were trained and given practice on the tasks, which involved constructing scenes in the imagination, perceiving visually presented scenes, or maintaining scenes in working memory. The prescan briefing began with an explanation of the tasks using pictures of the screens participants would later experience in the scanner (with stimuli not used in the scanning experiment) as visual aids. Participants then experienced 6 trials of the experiment while sitting at a desktop computer with the room lights extinguished, as it would appear in the scanner. To ensure they understood the task demands for scene and object construction, they then constructed 2 scenes and 2 objects out loud with the experimenter listening to ensure that they were performing the construction tasks correctly. They were then safety checked and taken to the scanner.

**Figure 1.**
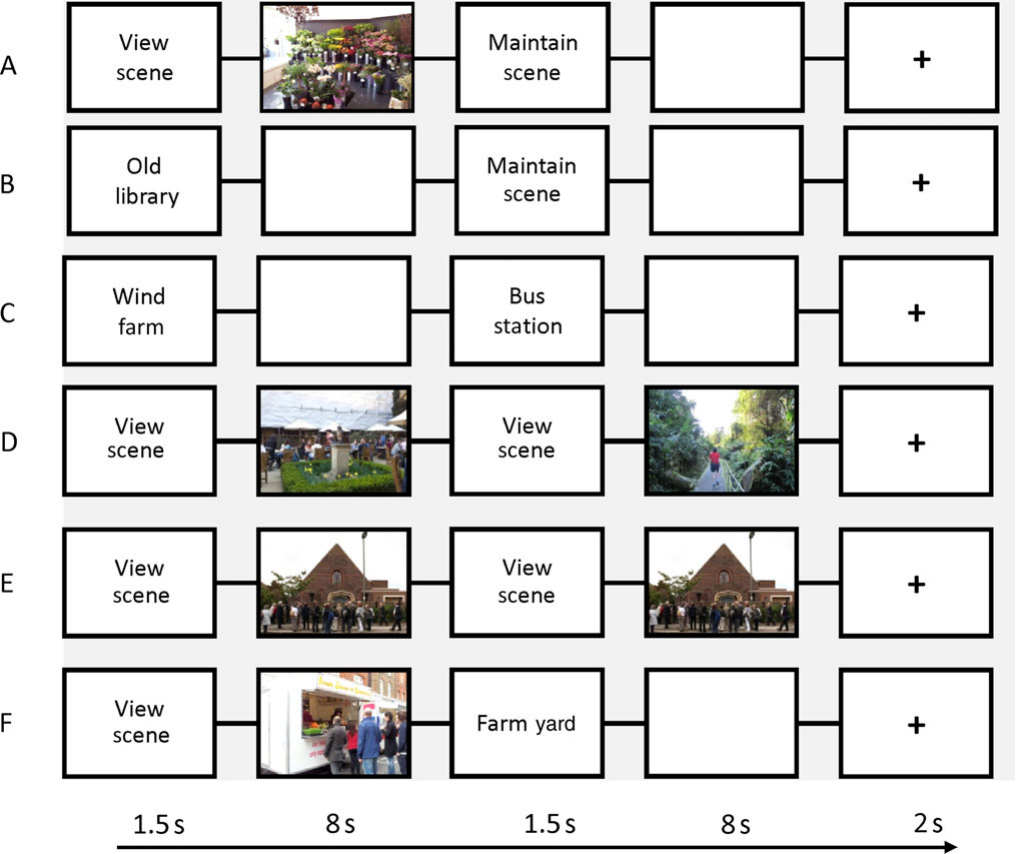
Scene trial types during the fMRI task. (*A*) Perceive then Maintain Scene—participants perceived a scene and then were cued to maintain that scene in their mind's eye while the screen went blank. (*B*) Construct then Maintain Scene—participants were given a two-word cue (e.g., “Old Library”), which indicated the scene they then had to construct in their mind's eye while the screen was blank. They were then cued to maintain that scene without changing anything. (*C*–*F*) The remaining trial types were Construct then Construct a different scene, Perceive then Perceive a different scene, Perceive then Perceive the same scene, and Perceive then Construct a different scene. For fMRI analysis, trials were divided into their first and second parts and collapsed to form regressors for perceive scenes, construct scenes, maintain perceived scenes, and maintain constructed scenes.

Each trial began with constructing or perceiving a scene. On some trials, the subject was then cued to maintain the scene in working memory, whereas other trials required the subject to perceive or construct a second scene. All trials ended with attending to a fixation cross. There were 6 types of scene trial, each with a different combination of constructing, perceiving, and maintaining scenes (Fig. 1).

Constructing scene trials began with a two-word cue (e.g., “Old library”) displayed for 1.5 s. Participants then created the scene in their imagination for the 8 s which followed, during which time the screen was blank. The duration of this period was determined by pilot testing to ensure subjects were comfortable performing the task. The prescan training emphasized that participants should make the scenes as vivid as possible and that they should keep their eyes open throughout. Use of episodic memory was discouraged, with subjects told “do not think of a scene you have experienced before, we want you to come up with something new.” The cues were designed to be of a similar nature to the scene types used in the perceive scene condition, such as “bus shelter,” “indoor pool,” and “hairdresser's salon.” Perceiving scene trials began with the cue “View Scene” displayed for 1.5 s, followed by a photograph of a scene displayed for 8 s. The scene photographs were selected to be an assortment of emotionally neutral indoor and outdoor, urban and rural scenes. For instance, they included a photograph of a typical UK high street, a park, and a bedroom. Participants were trained to perceive the scenes without attempting to recall episodic memories.

Having just constructed or perceived a scene, on some trials subjects saw the instruction “Maintain Scene.” They had been trained to hold a mental image of the most recent scene in their mind's eye, without changing anything or using their imagination.

Each scene condition had a matching object baseline condition. These were identical, except that subjects had to construct/perceive/maintain a single acontextual object alone against a plain white background. These were typical everyday objects, such as a shoe, a plant pot, or an apple. A simple vigilance task acted as a visual baseline. Participants were shown the cue “Pay attention,” after which a green cross appeared. They had to count the number of times the cross flashed red, which happened once, twice, or never in the course of a trial. Upon the fixation cross returning to black, participants indicated the number of flashes they saw using a keypad held in the right hand. There was one catch trial (where flashes actually occurred) per scanning session.

The full text of the training instructions is provided in Supplementary Text 1. Following training, participants underwent 3 consecutive scanning sessions, each around 16 min in length. For each participant, a total of 156 trials (72 scenes, 72 objects, 12 visual baselines) were presented in pseudo-random order and balanced across the 3 scanning sessions. Immediately following scanning, subjects completed a surprise memory test to evaluate their level of attention during the task. This involved a set of stimuli being shown to the participants, 33% of which were novel (lures) and the remainder were all the scene pictures, object pictures, and two-word scene and object construction cues used during scanning. Participants indicated with a button press whether they believed each stimulus was seen earlier, or was novel. Finally, the subjects were interviewed in order to collect ratings and to probe the cognitive strategies employed during the scanning tasks.

### Image Acquisition

MRI data were acquired on a 3T Magnetom Allegra head-only MRI scanner (Siemens Healthcare, Erlangen, Germany) operated with the standard transmit-receive 12-channel head coil. Functional MRI data were acquired in 3 sessions with a blood oxygenation level–dependent (BOLD)-sensitive *T*_2_*-weighted single-shot echo-planar imaging sequence which was optimized to minimize signal dropout in the medial temporal lobe (Weiskopf et al. 2006). The sequence used a descending slice acquisition order with a slice thickness of 2 mm, an interslice gap of 1 mm, and an in-plane resolution of 3 × 3 mm. Forty-eight slices were collected covering the entire brain, resulting in a repetition time of 2.88 s. The echo time was 30 ms and the flip angle 90°. All data were acquired at a −45° angle to the anterior–posterior axis. In addition, field maps were collected for subsequent distortion correction. These were acquired with a double-echo gradient echo field map sequence (TE = 10 and 12.46 ms, TR = 1020 ms, matrix size 64 × 64, with 64 slices, voxel size = 3 mm^3^) covering the whole head. After these functional scans, a 3D MDEFT *T*_1_-weighted structural scan was acquired for each participant with 1-mm isotropic resolution (Deichmann et al. 2004).

### Behavioral Data Analysis

Data from the post-scan memory test and interview were analyzed using repeated-measures ANOVAs and paired sample *t*-tests (SPSS 17.0, Chicago: SPSS, Inc.). The significance was set at *P* < 0.05.

### FMRI Data Analysis

Functional MRI data were analyzed using Statistical Parametric Mapping 8 (SPM8) (www.fil.ion.ucl.ac.uk/SPM). The first 6 “dummy” volumes from each of the 3 sessions were discarded to allow for *T*_1_ equilibration effects. Images were realigned and unwarped (using the field maps) and normalized to a standard template in MNI space using the DARTEL toolbox with a resampled voxel size of 3 × 3 × 3 mm.

The first analysis tested for a main effect of scenes across the whole brain. Data were smoothed using a 4-mm Gaussian kernel and entered into a general linear model (GLM) for each subject. Each trial was modeled as the onset of a cue with a duration of ∼9.5 s (i.e., each condition in Figure 1 was split into its 2 constituent parts). This formed 8 task regressors: construct scenes, perceive scenes, maintain constructed scenes, and maintain perceived scenes, plus each of their corresponding object baselines. Additional regressors removed the effects of head motion and catch trials in the visual baseline condition. To ensure that regressors would be uniquely identifiable, we used an experimental design with pseudo-randomized trial order. To validate the identifiability of the regressors, we examined the “design orthogonality” matrix produced by SPM for an example subject, which gives the cosine angle between all pairs of regressors in the model. The maximum (most collinear) absolute cosine angle between 2 task-related regressors was 0.15, with mean (non-zero) absolute cosine angle of 0.04. We were therefore satisfied that parameters relating to our conditions of interest were identifiable. Subject-specific parameter estimates pertaining to each regressor of interest (betas) were calculated for each voxel. Second-level random effects analyses were then run using one sample *t*-tests on these parameter estimates, collapsed across sessions. We report these results at a peak-level threshold of *P* <0.05 family-wise error (FWE) corrected.

To investigate our first 2 experimental questions—the relationship between perceiving and constructing scenes, and the relationship between constructing and maintaining scenes, we examined hippocampal responses within regions of interest (ROIs) for left hippocampus and right hippocampus. These were manually segmented from the averaged structural MRI scan of the 25 participants. We used an identical GLM as described above, except we used unsmoothed fMRI data to avoid activations encroaching from neighboring thalamus and PHC. Using SPM's corrected statistics on the unsmoothed data would have violated the smoothness assumptions of random field theory, and so significance thresholds were instead calculated at the second level using the nonparametric SnPM toolbox (http://go.warwick.ac.uk/tenichols/snpm). Results were thresholded at *P* <0.05 FWE-corrected for the small volume of bilateral hippocampus (minimum cluster size of 3 voxels). To quantify responses, we further subdivided the anatomical masks into anterior and posterior regions, with the most posterior slice of the uncus (*y* = −21) defined as the rear-most slice of anterior hippocampus. We then extracted the maximum response for each experimental condition within each mask and subtracted the object baseline responses from the scene responses. These results were then entered into a repeated-measures ANOVA and reported.

Our third experimental question concerned the functional connectivity between the hippocampus and the rest of the brain. We identified a region of overlap between scene perception and scene construction in each hippocampus, defined using a simple conjunction (logical AND) between FWE-corrected images for the contrasts: construct scenes–construct objects AND perceive scenes–perceive objects. The results of this conjunction (falling within the mask of bilateral hippocampi) were then divided into left and right hemispheres, and used as functional masks for the left and right ROIs. The signal from each functional mask was then extracted by taking the first eigenvariate (principal component) of the voxels' time series within that mask. We then examined their connectivity with the rest of cortex using the gPPI toolbox (McLaren et al. 2012). A gPPI analysis performs a similar function as a conventional PPI analysis, asking whether anywhere in the brain has a stronger relationship with the seed region during one condition compared with another (i.e., an interaction between the task and the time series of the seed region, regressed against every voxel in the brain using a GLM). The advantage of the gPPI approach over the standard PPI implementation is that it allows the experimenter to include all experimental conditions in a single model for a given seed region. A gPPI model was built and estimated for each ROI—one for the left hippocampal ROI and one for the right. Each model had PPI regressors for perceiving, constructing, maintaining perceived and maintaining constructed scenes, plus each of their object baselines (a PPI regressor being the time series of the ROI multiplied by the ON times of the conditions, which was then convolved with the canonical hemodynamic response function). Standard nuisance regressors for PPI analyses were also included (the seed region, the raw task regressors in addition to 6 motion regressors). Contrasts were calculated on these models for [(perceive scenes PPI – perceive objects PPI) – (construct scenes PPI – construct objects PPI)] and the reverse. The resulting contrast images were then summarized at the second level using one sample *t*-tests and displayed graphically on a “glass brain” with cerebella removed for clarity. Given the exploratory nature of the gPPI analyses, results were thresholded at *P* <0.001 uncorrected for multiple comparisons with a minimum extent of 5 voxels.

## Results

### Behavioral data

#### Attention to the Task

We tested attention during scanning using a simple incidental task. In the visual baseline condition, subjects were instructed to watch a green fixation cross and press a button to indicate whether it flashed red once, twice, or never. Three of 12 such trials were catch trials where flashes occurred, with one catch trial per scanning session. Subjects gave the correct response, on average, during 2.92 out of 3 catch trials (SD 0.28). There were no false-positive responses. We further evaluated attention to the task using a surprise post-scanning memory test. Subjects were shown a set of stimuli, two thirds of which were all the stimuli used during scanning (photographs from the perceive scene/object conditions, and text cues from the scene and object construct conditions), and one-third were lures. The task was to respond with “remember” or “don't remember” for each construct scene, construct object, perceive scene, and perceive object stimulus. Participants correctly identified 88.12% (SD 9.99%), 90.12% (SD 12%), 84.24% (SD 11.87%), and 86.56% (SD 12.49%) of stimuli, respectively. A repeated-measures ANOVA showed these results did not depend on whether the stimulus was a scene or object (*F*_1,24_ = 3.16, *P* = 0.09), whether constructed or perceived (*F*_1,24_ = 3.54, *P* = 0.07) or their interaction (*F*_1,24_ = 0.17, *P* = 0.90). A post hoc *t*-test confirmed no significant difference between the construct scenes and perceive scenes conditions (*t*_(24)_ = 1.87, *P* = 0.07). The false-positive rate was under 6% in all conditions.

#### Scene Construction Ratings

The “construct scene” condition required subjects to imagine novel scenes during scanning. The baseline condition “construct object” required subjects to imagine single novel objects, on their own without a surrounding scene or context. A series of post-scan ratings were used to evaluate subjects' performance on these tasks.

On a scale of 1–5 (maximum vividness = 5) subjects rated the vividness of the scenes and objects they constructed (scenes: mean 3.56, SD 0.71; objects: mean 3.96, SD 0.18) and this did not differ significantly for scene and object trials (*t*_(24)_ = 2.0, *P* = 0.06). They also rated the realism of their constructed scenes and objects (rating 1–5, completely realistic maximum = 5; scenes: mean 3.88, SD 0.83; objects: mean 4.48 SD 0.65), and this was significantly greater for objects than scenes (*t*_(24)_ = 3.133, *P* = 0.005). We also asked subjects about the content of their constructed scenes. They reported that on average 38.4% (SD 24.45%) of constructed scenes contained people, and 24.8% (SD 33.13%) implied movement. Fifty percent of stimuli used in the view scene condition contained people and 24% implied movement. There was no significant difference between constructed and perceived scenes for either measure of content (people: *t*_(24)_ = 0.31, *P* = 0.76; movement: *t*_(24)_ = 0.17, *P* = 0.87). Subjects were instructed to keep their imagined viewpoint within scenes in a fixed position and only construct what they would see in front of them (rating 1–5, maximum success at maintain a fixed viewpoint = 5; mean 3.56 SD 0.65). For the baseline object conditions, participants were instructed to imagine objects in isolation without a surrounding scene or context (rating 1–5, success at imagining in isolation maximum = 5; mean 3.92 SD 1.04).

#### Perceive Scene Ratings

Each “perceive scene” trial required subjects to passively study a photograph of a scene during scanning. To ensure separation from the construct conditions, it was important that subjects did not make significant use of their imagination. Subjects rated their use of imagination while looking at the scenes (rating 1–5, maximum use of imagination = 5: mean 1.60, SD 0.82), and the extent of their mind wandering during the task (rating 1–5, maximum occurrence of mind wandering = 5: mean 1.48 SD 0.82). These 2 ratings were not taken for perceiving object baselines. We also asked participants to describe how they spent their time during both the perceive scenes and perceive objects conditions, and we subsequently categorized these responses into those that suggested any imagination or self-projection with regards to perceived scenes/objects, and those which only indicated passive viewing. Twenty-one of 25 subjects reported passive strategies for scene viewing whereas 4 made mention of some interaction with the stimuli, for example “I felt … the temperature on the beach,” referring to a scene stimulus depicting a sandy beach. For object baselines, there were also 21/25 subjects who reported passive viewing strategies. The remaining 4 subjects, none of whom was the same as the 4 nonpassive subjects in the perceive scenes condition, included comments such as “I looked at details, imagined how it would feel—the textures.” While both ratings and descriptive measures showed that the majority of participants were not overtly using their imagination, we still confirmed that the 4 participants describing more active scene viewing were not unduly influencing our imaging results. We did this by re-analyzing our key fMRI analyses with only the 21 subjects reporting passive scene viewing and we found no differences in the results (Supplementary Text 2).

#### Maintain Scene Ratings

During the “maintain scenes' condition, subjects held an image of the most recently perceived or constructed scene in their mind's eye (and likewise for “maintain objects'). After scanning, participants reported their degree of success at maintaining the original stimuli without changing them (rating 1–5, maximum success at not changing items = 5: scenes mean 4.16, SD 0.16; objects mean 4.6, SD 0.1); although the success rating was high for both conditions, it was significantly higher for objects (*t*_(24)_ = 3.38, *P* = 0.003). Participants were also asked the question “How did you go about the maintain scenes task?” We sorted the responses according to whether subjects reported active processes, such as scanning the scene in their mind's eye, or instead gave a response that suggested passivity. Sixteen of 25 subjects reported active processes, such as “replaying details,” “tracing over,” and “drawing pictures with my eyes on the screen.” We confirmed that the fMRI results using data from just these 16 participants who described an active maintenance process were similar to the overall results which included the 25 subjects (see Supplementary Text 3).We also asked participants whether they experienced significant mind wandering during maintain conditions. Three participants answered “yes'” for maintaining scenes, and 4 answered “yes'” for maintaining objects, indicating most participants remained focused during the maintain tasks.

#### Other Ratings

Participants also rated the similarity to memories of the stimuli—constructed scenes (rating 1–5, identical to a memory = 5: mean 2.88, SD 0.83), perceived scenes (mean 2.2, SD 1.12), constructed objects (mean 2.84, SD 1.11), and perceived objects (mean 1.76, SD 1.13). A repeated-measures ANOVA revealed a main effect of task whereby construction was more similar to memories than perception (*F*_1,24_ = 16.22, *P* = 4.91e − 4). Importantly, however, the similarity to memories of constructed scenes did not differ significantly from the similarity to memories of its object baseline (*t*_(24)_ = 0.166, *P* = 0.87). Likewise, the similarity to memories of perceived scenes was not significantly different from the similarity to memories of its object baseline (*t*_(24)_ = 1.844, *P* = 0.08). The subtraction of the object baselines from the scene conditions in the fMRI analyses therefore goes some way toward controlling for the similarity to a memory effect.

Finally, we collected ratings to compare the difficulty of constructing scenes (rating 1–5, very difficult = 5: mean 2.4, SD 0.65), constructing objects (mean 1.32, SD 0.47), maintaining scenes (mean 2.36, SD 0.99), and maintaining objects (mean 1.68, SD 1.07). A repeated-measures ANOVA showed no difference in difficulty between constructing and maintaining scenes (*F*_1,24_ = 0.939, *P* = 0.34) but, as expected, scene conditions were significantly more difficult than their object baselines (*F*_1,24_ = 48.20, *P* = 3.53e − 7). We also separately examined the 9 of 25 subjects who did not report the maintain scenes condition as being active. We found that their difficulty ratings for maintaining scenes (mean 2.44, SD 0.72) were in fact not significantly different from the difficulty ratings they reported for constructing scenes [mean 2.67, SD 0.5; *t*-test (*t*_(8)_ = 1.51, *P* = 0.17)].

In summary, our post-scan ratings showed good adherence with the task instructions across subjects, and demonstrated that the scenes were matched for content (people and movement) across conditions, while scenes and object baselines were matched for vividness and similarity to memory. Constructed objects had greater realism than constructed scenes, although we still considered the rating for scenes to be high (mean 3.88 of 5, SD 0.83). Participants were also better at maintaining objects than maintaining scenes without changing them, although the difference was marginal and success for scenes was rated as 4.16 of 5 (SD 0.16). Finally, constructing scenes was rated as significantly more difficult than constructing single isolated objects, and we suggest that this added difficulty was taxing the cognitive processes of interest.

### fMRI data

We begin with a summary of responses across the whole brain to constructing, perceiving, and maintaining scenes before focusing on our ROI, the hippocampus.

#### Confirming Engagement of the Scene Network

**Figure 2.**
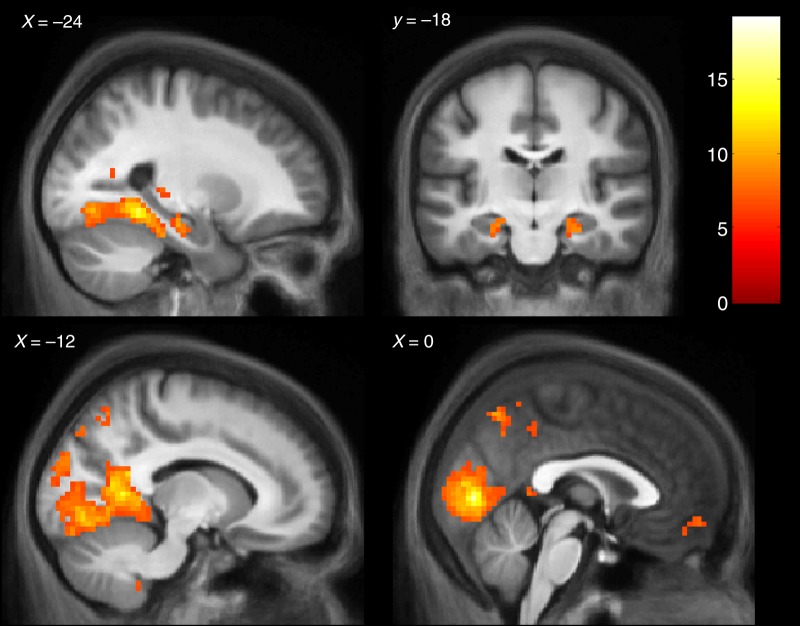
Main effect of scenes. Top-left: sagittal slice displaying left anterior medial hippocampus and PHC. Top-right: Coronal slice displaying anterior medial hippocampus. Bottom-left: Sagittal slice displaying left RSC and medial occipital cortex. Bottom-right: Sagittal slice displaying ventromedial prefrontal cortex, precuneus and medial occipital cortex. Calculated using the contrast of scene conditions relative to their individual object baselines ([perceive scenes + construct scenes + maintain perceived scenes + maintain constructed scenes] − [perceive objects + construct objects + maintain perceived objects + maintain constructed objects]). Thresholded at *P* < 0.05 FWE-corrected and displayed on the averaged structural MRI scan of the 25 subjects. The color bar indicates the *t*-statistic. See Table 1 for a full description of the results.

We confirmed that our stimuli engaged brain regions known to respond to scene construction, which previous studies had shown to include hippocampus, PHC, RSC, and medial prefrontal cortex (Hassabis, Kumaran, and Maguire 2007; Spreng et al. 2009; Summerfield et al. 2010). A contrast was calculated of the scene conditions relative to the object baselines ([perceive scenes + construct scenes + maintain perceived scenes + maintain constructed scenes] − [perceive objects + construct objects + maintain perceived objects + maintain constructed objects]), which revealed activation of a priori scene regions in addition to a large area of visual cortex (Fig. 2, Table 1). We noted that the activation did not cover the entirety of the hippocampus, but rather was situated in a circumscribed anterior region, in the medial rather than lateral portion bilaterally (Fig. 2, top row). Having confirmed these predicted activations at the whole-brain level, we next looked within ROIs of the hippocampi to address our specific experimental questions.

**Table 1:** Activation peaks for the contrast of Constructing, Perceiving, and Maintaining Scenes—Objects baselines

*X*, *Y*, *Z*	*Z*	Region
Scenes—Objects		
−3, −78, 3	>7.38	L Calcarine sulcus
−21, −45, −9	7.38	L Parahippocampal cortex
−12, −54, 6	7.19	L Retrosplenial cortex
24, −36, −15	7.05	R Parahippocampal cortex
6, −42, 6	7.03	R Retrosplenial cortex
27, −18, −21	5.77	R Anterior medial hippocampus
45, −63, 21	6.28	R Occipital gyrus
−24, −21, −18	6.21	L Anterior medial hippocampus
−3, −63, 54	6.15	L Precuneus
−33, −81, 30	6.10	L Occipital gyrus
18, −42, −48	5.74	R Cerebellum
0, 57, −12	5.38	Ventromedial prefrontal cortex
−54, −12, −18	5.32	L Superior temporal sulcus
6, −54, 66	5.18	R Precuneus
−27, 18, 54	5.16	L Superior frontal sulcus
−3, −42, 48	5.16	L Precuneus
21, 0, 54	5.15	R Superior frontal sulcus
−6, −51, −39	5.10	L Cerebellum
−3, −54, 63	5.02	L Precuneus
−12, −45, −48	5.01	L Cerebellum
3, −51, −39	4.98	R Cerebellum
54, −6, −15	4.87	R Superior temporal sulcus

Note: Thresholded at *P* < 0.05 FWE-corrected for the volume of the whole brain, minimum extent 2 voxels. The first cluster subsumed 5 regions expected a priori, and these subpeaks are expanded for clarity.

L, left; R, right.

### Hippocampal ROIs

#### Location of Responses

**Figure 3.**
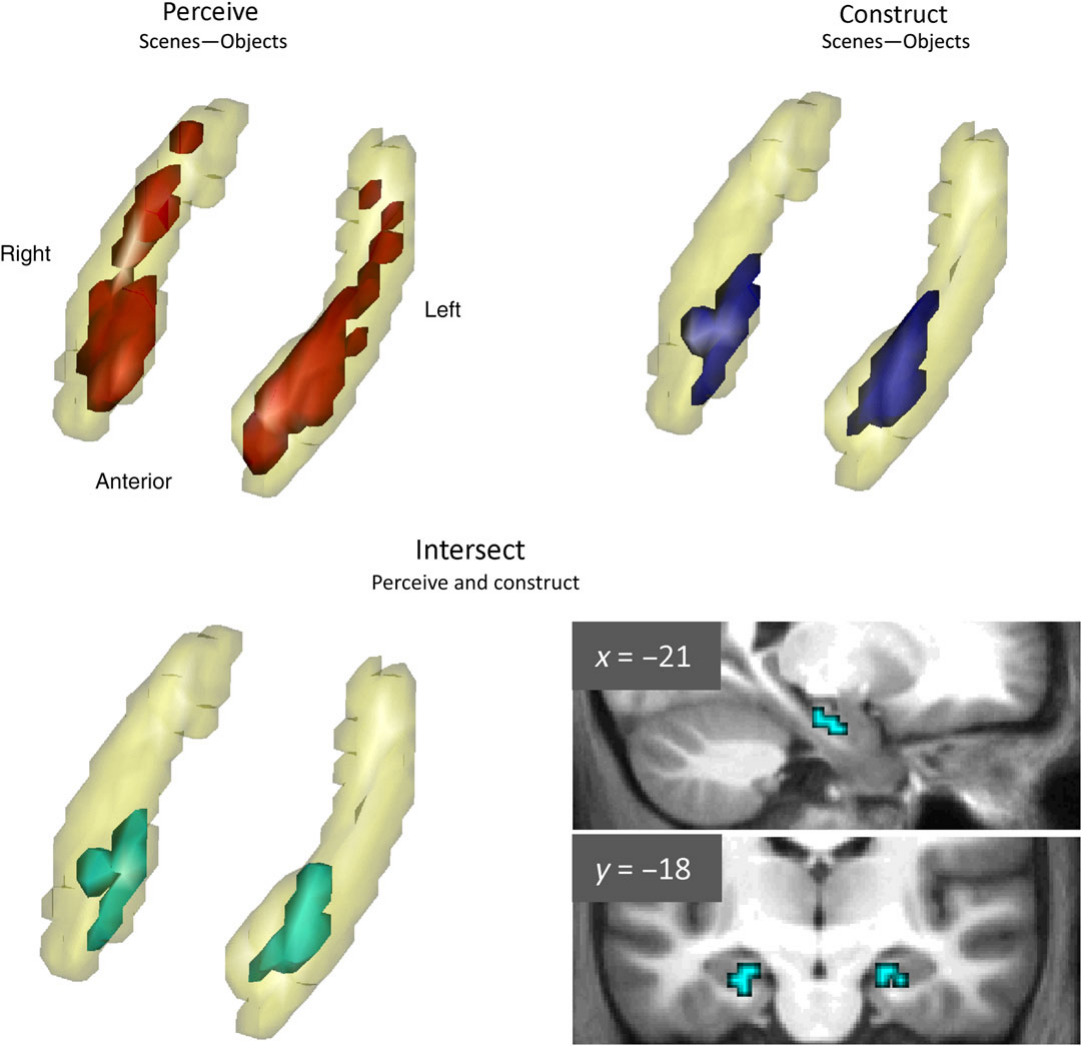
ROI analysis within a mask of bilateral hippocampus. Top-left: Activation for perceiving scenes, relative to the matched baseline of perceiving objects, shown on a 3D projection of the hippocampus. The viewpoint is at the front of the brain looking down. Top-right: Activation for constructing scenes, relative to the matched baseline of constructing objects. Bottom-left: Voxels significantly engaged by both scene perception and scene construction, shown in 3D projection. Bottom-right: The intersection shown on sagittal and coronal slices of the group average structural MRI scan, thresholded at *P* < 0.05 FWE-corrected for the bilateral hippocampal mask. See the text for a full description of the results.

We began by examining the spatial distribution of voxels responding to our experimental conditions within the hippocampus. We performed group-level SPM analyses with FWE-corrected statistics for the small volume of bilateral hippocampus. The contrast of perceiving scenes relative to perceiving single acontextual objects (perceive scenes – perceive objects) revealed a swathe of voxels along the length of bilateral hippocampi (Fig. 3, top left), with peak coordinates at (−21, −12, −21) in left and (30, −30, −6) in right hippocampus. Constructing scenes (construct scenes – construct objects) engaged a cluster of voxels only in anterior medial hippocampi (Fig. 3, top right), with peaks at (−21, −18, −21) in left and (24, −15, −21) in right hippocampi, respectively. To identify voxels involved with scene maintenance, we performed the contrast of maintain constructed scenes – maintain constructed objects. No voxels were significantly activated in this contrast. Although not the main focus of this investigation, we also examined the maintenance of perceived scenes. This contrast (maintain perceived scenes – maintain perceived objects) found only one voxel in left hippocampus significantly responding to maintaining perceived scenes (−21, −21, −15). Calculating the intersection (logical conjunction) of the activation for perceiving and constructing scenes showed that only voxels in anterior medial hippocampi were engaged by both conditions (Fig. 3, bottom left and right), pointing to the possibility that this subregion might be performing a common cognitive process. Using the hippocampal delineation scheme of Poppenk et al. (2013), who suggested defining the rear of the uncus as the border of anterior–posterior hippocampus, these activations were located within anterior hippocampus (rear-most voxel *y* = −21). For completeness, the results of these contrasts (and those in the next section) across the whole brain are provided in Supplementary Tables 1 and 2.

#### Comparison of Conditions

To quantify and compare these hippocampal responses, we created masks covering left anterior, right anterior, left posterior, and right posterior hippocampus (see Materials and Methods). For each subject, peak parameter estimates were extracted within each mask for each scene condition and object baselines. The betas for the object conditions were subtracted from the corresponding scene conditions, and the results entered into a repeated-measures ANOVA with factors of hemisphere (left, right), region (anterior, posterior), and condition (perceive, construct, maintain perceived, maintain constructed).

**Figure 4.**
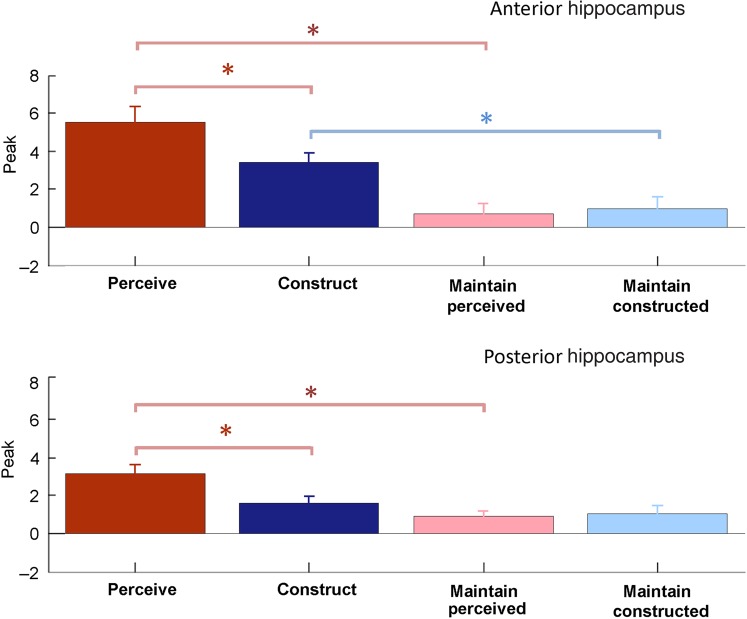
Summary and comparison of ROI results. Top: Anterior hippocampus (from the most anterior slice to *y* = −21 inclusive). Bottom: Posterior hippocampus. Bars represent the peak parameter estimate for the relevant scene condition minus the peak parameter estimate for the relevant object baseline. Results are collapsed over hemisphere. Error bars indicate standard error across subjects and asterisks indicate significant differences based on paired sample *t*-tests at *P* < 0.05; see text for full results.

There was no significant effect of hemisphere (*F*_1,24_ = 0.477, *P* = 0.497) nor any interactions involving hemisphere. For this reason, the ROI results (Fig. 4) are shown collapsed over hemisphere. There was a significant main effect of region (anterior/posterior) (*F*_1,24_ = 6.174, *P* = 0.02), and Figure 4 shows that this was driven by the stronger response in anterior than posterior hippocampus to the perceive and construct conditions. There was a significant main effect of condition (*F*_3,72_ = 22.105, *P* = 2.98e − 10) and a significant interaction between condition and region (*F*_3,72_ = 3.989, *P* = 0.018).

To unpack the effect of experimental condition on hippocampal response, we consider our first experimental question concerning the relationship between scene perception and scene construction. Paired *t*-tests showed the responses were significantly greater for scene perception than construction in both anterior (*t*_(24)_ = 2.91, *P* = 0.008) and posterior hippocampi (*t*_(24)_ = 3.86, *P* = 7.42e − 4). Our second question was whether scene maintenance would be sufficient to explain the hippocampal response to scene construction. In anterior hippocampus, the response was significantly greater for constructing scenes than maintaining constructed scenes (*t*_(24)_ = 5.05, *P* = 3.69e − 5). Reflecting the SPM result that scene construction was limited to anterior hippocampus, there was no significant difference between constructing and maintaining constructed scenes in posterior hippocampus (Fig. 4, bottom; *t*_(24)_ = 1.00, *P* = 0.33). Similarly, there was a significantly greater response to perceiving scenes than maintaining perceived scenes in anterior (*t*_(24)_ = 4.71, *P* = 8.17e − 5) as well as in posterior (*t*_(24)_ = 4.45, *P* = 1.68e − 4) hippocampus. There was no significant difference between maintaining perceived and maintaining constructed scenes in anterior (*t*_(24)_ = 0.36, *P* = 0.72) or posterior (*t*_(24)_ = 0.30, *P* = 0.77) hippocampus.

We conducted 2 supplementary analyses. In the first (Supplementary Table 3), we tested whether these responses correlated with subsequent memory for the stimuli. There were no significant correlations in any part of the hippocampus with subsequent memory. In the second (Supplementary Text 4 and Figs 1 and 2), we separated out perceiving novel from perceiving repeated scenes, conditions which were collapsed in all other analyses presented here. There was a stronger response in anterior hippocampus to scenes than objects when the stimuli were novel. In contrast, posterior hippocampus responded more strongly to scenes than objects regardless of novelty.

#### Hippocampal Connectivity

We next performed an exploratory investigation into hippocampal connectivity with wider brain areas using gPPI, a technique which identifies locations in the brain correlated with a seed region in the context of one experimental condition over another. We included the left and right anterior medial hippocampal regions that were engaged in common by constructing and perceiving scenes as seed regions in the PPI analysis. We then compared the connectivity between the hippocampus and other brain regions for perceiving scenes, constructing scenes, and constructing relative to perceiving scenes (with object baselines subtracted). Maintaining scenes was not included in this PPI analysis because without significant activation of the hippocampus in this condition, the choice of voxels to include in the seed region would have been difficult to motivate.

Perceiving scenes relative to objects (Table 2) lead to greater connectivity between right hippocampus and bilateral PHC and right occipito-parietal junction. Left hippocampus was associated with the same regions, plus left occipito-parietal junction, right RSC, and cerebellum. Constructing scenes relative to objects (Table 2) engaged greater connectivity between right hippocampus and right PHC, left orbito-frontal cortex, right RSC, left fusiform gyrus, left superior frontal gyrus, right precuneus, and primary visual cortex. Left hippocampus connected with right PHC, left superior temporal sulcus, left occipito-parietal junction, right RSC, and bilateral superior frontal gyrus.

**Table 2:** Hippocampal network for construct scenes > objects and perceive scenes > objects (PPI)

*X*, *Y*, *Z*	*Z*	Region	Hippocampus seed
Construct Scenes > Objects (PPI)			
30, −39, −9	5.50	R PHC	Left
−48, −12, −15	3.93	L STS	Left
−18, −57, 12	4.36	L OPJ	Left
15, −48, 9	3.66	R OPJ	Left
3, 12, 51	3.60	R Superior frontal gyrus	Left
−6, 15, 51	3.52	L Superior frontal gyrus	Left
30, −36, −12	4.81	R PHC	Right
−39, 33, −18	4.27	L OFC	Right
6, −42, 3	4.04	R RSC	Right
−12, −54, 15	3.61	L OPJ	Right
−30, −36, 24	3.60	L Fusiform gyrus	Right
−6, 15, 51	3.53	L Superior frontal gyrus	Right
6, −66, 48	3.43	R Precuneus	Right
15, −84, 0	3.37	R Calcarine sulcus	Right
Perceive scenes > objects (PPI)			
30, −36, −15	4.24	R PHC	Left
18, −57, 21	4.04	R RSC	Left
−27, −42, −15	3.96	L PHC	Left
0, −54, −33	3.85	Cerebellum	Left
−15, −39, −45	3.78	Cerebellum	Left
−18, −54, 6	3.40	L OPJ	Left
−27, −42, −15	4.31	L PHC	Right
21, −30, −21	4.24	R PHC	Right
21, −57, 18	3.51	R OPJ	Right

Note: Thresholded at *P* < 0.001 uncorrected, minimum extent 5 voxels.

OPJ, occipito-parietal junction; STS, superior temporal sulcus; L, left; R, right.

**Figure 5.**
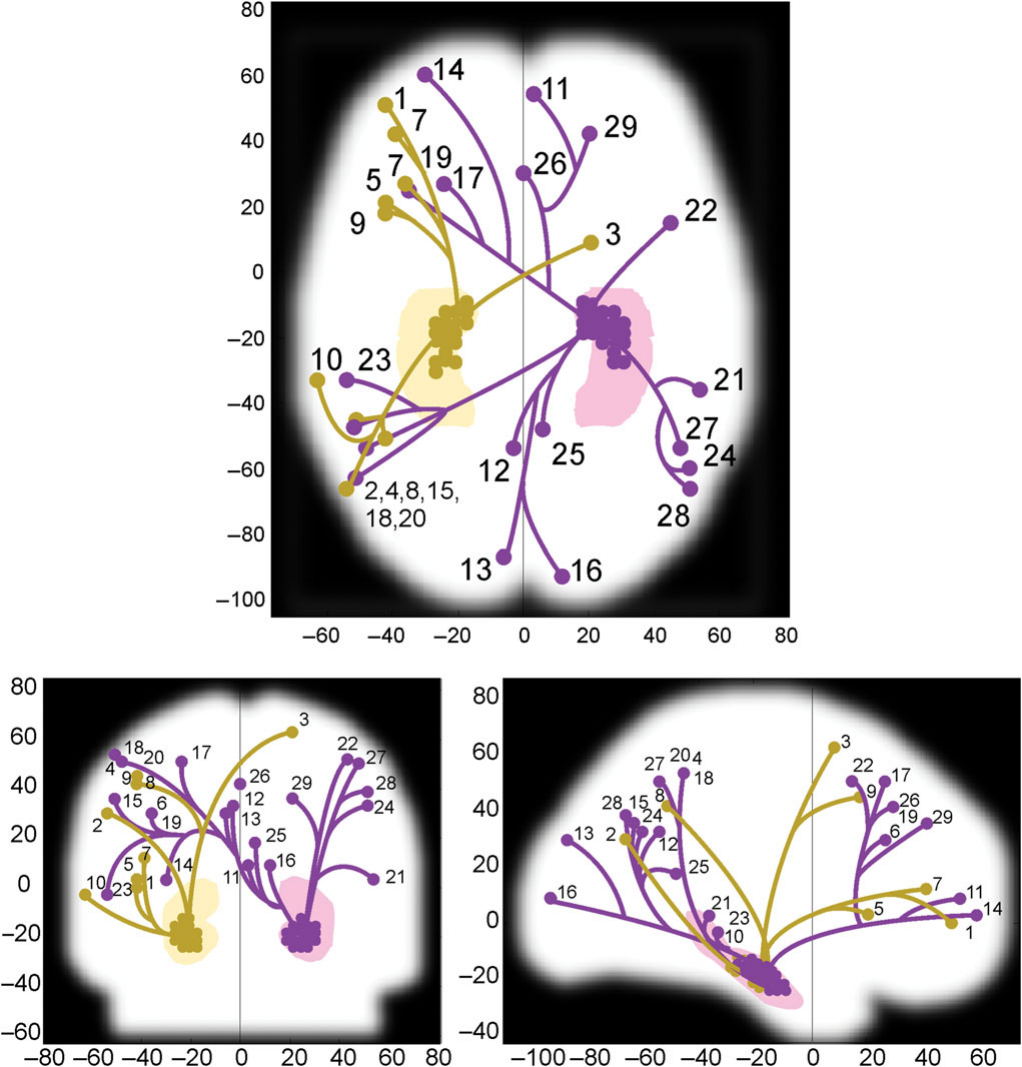
Exploratory PPI analysis of constructing more than perceiving scenes, with object baseline conditions subtracted from each. Projections (shown in the axial, coronal, and sagittal planes) of the left anterior medial hippocampal region are shown in gold and the right anterior medial hippocampal region in purple. Small circles outside the hippocampi indicate peaks of activation which had significantly stronger connectivity with the corresponding hippocampus for constructing than perceiving scenes. The branching pattern is for visual clarity only. Numbers refer to regions detailed in Table 3. The left side of the brain is on the left. There were no significant PPI results for the reverse contrast of Perceive > Construct Scenes. Both contrasts were conducted at *P* < 0.001 uncorrected with a minimum extent of 5 voxels.

Next, we directly compared the connectivity associated with perceiving and constructing scenes (with object baselines subtracted). No brain region had greater connectivity with the hippocampus for perceiving more than constructing scenes. However, the reverse contrast of constructing more than perceiving scenes revealed increased hippocampal connectivity with a wide set of brain regions (Fig. 5, Table 3), despite the univariate SPM analysis not finding any voxels with a greater activation for scene construction than scene perception.

**Table 3:** Hippocampal network for construct > perceive scenes (PPI)^a^

	*X*, *Y*, *Z*	*Z*	Region	Hippocampus seed
1	−42, 51, 0	4.54	L DLPFC	Left
2	−54, −66, 30	4.18	L Posterior parietal cortex	Left
3	21, 9, 63	4.15	R Superior frontal gyrus	Left
4	−51, −45, 54	3.94	L Supramarginal gyrus	Left
5	−42, 21, 3	3.88	L Supramarginal gyrus	Left
6	−36, 27, 30	3.82	L Middle frontal gyrus	Left
7	−39, 42, 12	3.78	L Inferior frontal sulcus	Left
8	−42, −51, 42	3.76	L Supramarginal gyrus	Left
9	−42, 18, 45	3.41	L Middle frontal gyrus	Left
10	−63, −33, −3	3.4	L Superior temporal sulcus	Left
11	3, 54, 9	4.22	R Medial PFC	Right
12	−3, −54, 33	4.16	L Precuneus	Right
13	−6, −87, 30	4.04	L Dorsal occipital gyrus	Right
14	−30, 60, 3	4.02	L Superior frontal gyrus	Right
15	−51, −63, 36	3.99	L Angular Gyrus	Right
16	12, −93, 9	3.98	R Calcarine sulcus	Right
17	−24, 27, 51	3.98	L Superior frontal sulcus	Right
18	−51, −45, 54	3.9	L Inferior parietal lobule	Right
19	−36, 27, 30	3.86	L Middle frontal gyrus	Right
20	−48, −54, 51	3.85	L Inferior parietal lobule	Right
21	54, −36, 3	3.79	R Superior temporal sulcus	Right
22	45, 15, 51	3.75	R Middle frontal Gyrus	Right
23	−54, −33, −3	3.73	L Superior temporal sulcus	Right
24	51, −60, 33	3.68	R Angular gyrus	Right
25	6, −48, 18	3.63	R Posterior cingulate cortex	Right
26	0, 30, 42	3.57	Anterior cingulate cortex	Right
27	48, −54, 51	3.53	R Inferior parietal lobule	Right
28	51, −66, 39	3.5	R Inferior parietal lobule	Right
29	21, 42, 36	3.46	R Superior frontal sulcus	Right

Note: Thresholded at *P* < 0.001 uncorrected, minimum extent 5 voxels.

DLPFC, dorsolateral prefrontal cortex; L, left, R, right.

^a^See also Figure 5.

Most of the PPI results were observed using the right rather than the left hippocampus as the seed region. In addition, the right hippocampal activation was associated with activity in regions of both hemispheres, while the left hippocampal activation was mainly associated with activity in ipsilateral brain areas. When participants constructed scenes, there was stronger connectivity between right hippocampus and occipital cortex, with clusters including primary visual cortex (dorsal to the calcarine fissure) and dorsal medial occipital cortex, immediately posterior to the occipito-parietal junction. Based on their position, these likely correspond to areas V1 and V6, respectively (Pitzalis et al. 2006). In prefrontal cortex, there were clusters in anterior medial and dorsolateral prefrontal cortex, the latter also being found when using left hippocampus as the seed. Parietal cortex activation was also associated with activity in both hippocampi for constructing scenes, including overlapping clusters in left inferior parietal lobule (IPL). Medially, there was connectivity between precuneus, posterior cingulate cortex, and right hippocampus. Temporal cortex contained clusters in bilateral superior temporal sulci, with right hippocampus as the seed.

Together, these findings demonstrated substantially different functional connectivity of the hippocampus depending on whether scenes were constructed or perceived, with stronger connectivity between anterior medial hippocampus and a distributed set of brain regions when constructing than perceiving scenes. Additionally, we tested for differences in connectivity between posterior hippocampus, which was only engaged by the perceive scenes condition, and the rest of the brain (Supplementary Text 5). Each posterior hippocampus had significantly stronger connectivity with contralateral PHC when perceiving scenes relative to objects. Further PPI analyses in posterior collateral sulcus, a region on the occipital/temporal cortex border which was activated in the perceive scenes – perceive objects contrast, revealed stronger connectivity with both primary visual cortex and PHC when perceiving scenes relative to single objects. These results suggest that posterior hippocampus and early visual cortex interact when viewing scenes, via posterior collateral sulcus and PHC.

## Discussion

We set out to characterize the response and connectivity of the hippocampus when perceiving scenes, constructing scenes in the imagination and maintaining scenes in working memory. We first asked whether scene perception would engage the hippocampus, and if so, how this would compare to scene construction. We observed the involvement of the hippocampus in both perceiving and constructing scenes. Perceiving scenes gave rise to extensive activation along the length of bilateral hippocampi, whereas scene construction was restricted to anterior hippocampus, and we identified a region of anterior medial hippocampus common to both. Second, we asked whether maintaining scenes would be sufficient to explain the involvement of the hippocampus in scene construction. Comparing scene conditions revealed that maintaining scenes elicited significantly less activity within the hippocampus than constructing or perceiving scenes. Third, we explored the connectivity of the hippocampus during scene construction and scene perception, and found significantly stronger connectivity between anterior medial hippocampus and a distributed set of brain areas during scene construction compared with scene perception.

### Scene Perception and the Posterior Hippocampus

Whereas scene construction engaged only the anterior medial portion of the hippocampus, scene perception engaged both posterior and anterior hippocampus. We begin by considering our findings in posterior hippocampus. The differences we observed in this region were unlikely to be due to differences in the nature of the scenes used in our perceive and construct conditions, as extensive pilot testing ensured similarities in content and detail. Rather, our results suggest posterior hippocampus may have a particular role in scene perception. Previous studies have demonstrated engagement of posterior hippocampus in tasks involving the discrimination of visually similar scenes (Lee, Buckley, et al. 2005; Lee, Bussey, et al. 2005, 2008, 2012; Barense et al. 2010; Mundy et al. 2012; Aly et al. 2013). Our finding builds on the scene discrimination literature in 2 respects. First, these studies did not distinguish scene construction (creating an internal representation of the scene to facilitate discrimination) from processes which are specific to visual perception. In this study, we used a within-subjects design to directly compare visual scene perception against scene construction without visual perception. We found that scene construction was not sufficient to engage posterior hippocampus, but it was engaged when driven by visual stimuli. Second, in this study, we demonstrated engagement of the hippocampus during scene viewing without explicit task demands, which may better reflect its function in everyday perception than when tested using discrimination tasks.

Although we treated perception and construction as separate conditions, we do not suggest that scene perception and construction are independent—as evidenced by the overlap between perception and construction in anterior hippocampus. We propose that a common feature of perception and construction is the need to form an internal model of the scene, and this scene construction process is mediated by anterior medial hippocampus (we return to anterior hippocampus in the next section). Given this hypothesis, what function(s) may be performed by posterior hippocampus which explain why it was engaged by perceiving scenes relative to perceiving single objects?

One possibility is that posterior hippocampus processes visuospatial input to support the formation of a scene representation. This is in keeping with previous studies associating spatial processing with dorsal hippocampus in rats (Jung et al. 1994; Moser and Moser 1998) and posterior hippocampus in humans (Maguire et al. 2000, 2006) and monkeys (Colombo et al. 1998). Indeed, the anatomical connectivity of posterior hippocampus gives good reason to expect a particular role in visual scene perception. It has strong connectivity with the ventral visual streams via PHC (Kahn et al. 2008; Libby et al. 2012), and a supplementary PPI analysis of our data (Supplementary Text 5) suggested that visuospatial sensory information may have reached posterior hippocampus via PHC. Constructed scenes, in contrast, were endogenously generated and so did not contain primary sensory input. Further evidence that posterior hippocampus supports scene construction when viewing scenes comes from recent work on scene discrimination. Aly and Yonelinas (2012) distinguished 2 processes used by participants to perform scene discrimination—making judgments based on the scene's global configuration, or strength-based perception, and based on local visual features, or state-based perception. Strength-based perception depends on constructing a model or representation of the global features of the scene—and the authors identified left posterior hippocampus associated with this process (Aly et al. 2013).

The role of posterior hippocampus may go beyond processing the visuospatial information currently available and extend to making predictions of the scene beyond the view. Posterior hippocampus has been implicated in BE, the automatic process of extrapolating beyond the view (Intraub and Richardson 1989). Patients with hippocampal lesions showed attenuated BE (Mullally, Intraub, and Maguire 2012) and Chadwick et al. (2013) found that specifically posterior hippocampus was engaged when healthy controls viewed simple scenes and experienced BE. Thus, posterior hippocampus may contribute predictions of what lies beyond the edges of the current view, to support the creation and updating of a scene model in anterior medial hippocampus. In a supplementary analysis using a subset of our data, we compared the perception of novel scenes with the perception of scenes which had already been viewed for 8 s (Supplementary Figs 1 and 2 and Text 4). We found that posterior hippocampus was engaged when perceiving repeated scenes as well as scenes that were novel. In contrast, anterior hippocampus was only engaged when scenes were novel, relative to object baselines. This finding is consistent with a role for posterior hippocampus constantly predicting beyond the boundaries of the scene, regardless of scene novelty. A related possibility is that visuospatial predictions from posterior hippocampus not only support the creation of an internal model of the scene, but also guide ongoing scene perception. Bayesian and predictive coding theories of perception suggest that higher brain areas send predictions down the sensory processing hierarchy (Jaynes 1988; Friston and Kiebel 2009), and the benefit of having a scene model to guide perception has long been recognized. By playing a role in setting up expectations, also called contexts, schemas (Biederman 1981), gist or frames (Friedman 1979), it is suggested that scenes facilitate the subsequent identification of objects (reviewed by Henderson and Hollingworth 1999). Further experiments will be needed to test whether prior knowledge of a scene modulates posterior hippocampus activity.

An alternative explanation for our findings is that posterior hippocampus simply functions to encode a memory of the scene. When stimuli are being processed which exceed the limit of working memory, the hippocampus may be engaged to encode stimuli in LTM (Jeneson and Squire 2012). Furthermore, as our participants did not know if they would subsequently have to maintain the scenes they perceived, they may have been making a particular effort to remember their details. Several lines of evidence suggest that LTM encoding is not sufficient to explain our results in posterior hippocampus. First, each scene was compared against a matched baseline of perceiving single objects. A surprise memory test after scanning showed that objects were remembered equally well as the scenes, and so these baselines should have accounted for basic encoding processes. However, it could be argued that the hippocampal preference for scenes or complex stimuli means that it would be involved in encoding scenes but not objects into LTM and, thus, the object baselines would not control for this. One way to de-correlate hippocampal scene perception from memory encoding would be to look at subsequent memory effects. A previous study with a similar time interval as ours specifically investigated the relationship between scene perception in the hippocampus and subsequent memory. Lee et al. (2013) found hippocampal activation for scene oddity judgments, regardless of whether participants subsequently remembered or forgot the scenes 10 min later. Indeed, in an additional analysis of our data, we found no correlation between hippocampal activation and subsequent memory performance. Furthermore, if memory encoding were responsible for activation of posterior hippocampus, one might expect that perceiving a repeated or familiar scene would not engage posterior hippocampus, as there would be nothing new to encode. As already mentioned, we compared perception of novel with familiar scenes. While there was a significant novelty response in posterior hippocampus, both novel and repeated scenes gave robust activation, arguing against encoding being sufficient explanation for our posterior hippocampus activation.

While our main interest in including scene maintenance was to compare it against scene construction, our results also speak to a related question—whether hippocampal engagement in scene perception may be explained by maintenance. This is of particular importance for considering the results of scene discrimination studies, where multiple scenes must be held online while they are compared. Lee and Rudebeck (2010) found that working memory load increased right posterior hippocampal response to scenes, but not baseline shape stimuli. Other studies have also suggested hippocampal engagement in scene memory over short retention periods (Hannula et al. 2006; Schmidt et al. 2007; Jeneson et al. 2011). In this study, we tested perceiving scenes, maintaining perceived scenes in working memory and compared the 2 and found only a single significant voxel in left hippocampus for scene maintenance, whereas there was extensive activity throughout bilateral hippocampi for scene perception, and the activity for perception was significantly stronger than for maintenance. Thus, the need to maintain the contents of the scene while it is perceived does not seem to explain hippocampal activation for perception. It is pertinent to consider how this maintenance result relates to previous studies. First, we focused specifically on the working memory maintenance period, unlike the other studies referenced above, and we also did not manipulate memory load. A further difference concerns the task demands. The study by Lee and Rudebeck (2010), for instance, required participants to compare the spatial location of elements of the currently viewed scene against scenes they had previously viewed (a 1- or 2-back test). Unlike their study, our participants only had to maintain one scene at a time and so did not need to switch between perceived and remembered scenes during a trial. This switching effect could tax the same construction process of interest here. More generally, adding more scenes or adding more detail within scenes to increase working memory load may also place greater demand on the processes which construct the hippocampal representation of the scenes. Thus, care should be taken when attempting to separate working memory load from the scene construction process.

In reviewing anterior–posterior differences in the hippocampus, Poppenk et al. (2013) suggested that posterior hippocampus responds to fine detail or fine-grained representations, whereas anterior hippocampus processes more coarse or abstract representations. One aspect of “detail” may be the number of objects which make up the scene, and perhaps the perceived scenes in our study had more objects than the constructed scenes, leading to additional posterior hippocampal engagement. Although we did not take per-trial ratings of the level of detail, our imagined scenes were rated high on realism and we established that aspects of the content did not differ significantly between constructed and perceived scenes. In addition, both scene types were recalled with similar accuracy in a post-scan memory test. Furthermore, the study of Chadwick et al. (2013), which associated BE with engagement of posterior hippocampus, used stimuli consisting of very simple scenes (a single object on a naturalistic background) displayed for 250 ms, suggesting even scenes containing a single object are sufficient to engage posterior hippocampus. A second aspect of detail may concern fine-grained visual or spatial detail, which could vary independently of the number of objects. Participants' ratings for vividness were matched for perceived and constructed scenes, although again this was not a per-trial measure and imagined objects within the constructed scenes may have had less detail than objects in the scene photographs. Thus, fine-grained visual information could have contributed to the response we observed in posterior hippocampus for the perceive scenes condition. However, we note that our “perceive object” baseline condition, which consisted of color photographs of objects, should have controlled for detail to some extent. Furthermore, studies varying the spatial frequency of visually presented stimuli, and thereby controlling the level of fine detail, did not identify a difference in hippocampal response (Rajimehr et al. 2011; Zeidman et al. 2012).

In summary, we found posterior hippocampus to be involved in scene perception. We suggest that it contributes to the construction of a coherent model of the scene by processing visuospatial input and that memory encoding or detail are not sufficient to explain its role. The anterior–posterior hippocampal distinction we have documented here adds to a growing body of evidence of functional differentiation within the hippocampus that clearly requires further elucidation (Poppenk et al. 2013).

### Scene Perception and Scene Construction Engage the Anterior Medial Hippocampus

We found that visually perceiving scenes and constructing scenes in the imagination engaged a common brain region, located in the medial aspect of anterior hippocampus. What function might this region serve? Of note, other fMRI studies involving scene stimuli have also implicated anterior medial hippocampus, with tasks such as imagining and recalling scenes (Hassabis, Kumaran, and Maguire 2007), spatial and autobiographical memory recall (Hoscheidt et al. 2010), and scene discrimination (Lee et al. 2013).

One possibility is that anterior hippocampus forms arbitrary associations between stimuli (Schacter and Wagner 1999). While the process of scene construction involves forming associations between elements of the scene representation across cortex (Maguire and Mullally 2013), and as evidenced by our connectivity results which we return to shortly, we suggest that it is unlikely that the primary function of anterior hippocampus is to encode arbitrary associations. Patients with bilateral hippocampal damage can state the objects they would expect to see beyond the current view, suggesting preserved processing of associations, but they cannot describe the spatial relationships between objects they cannot see (Mullally, Intraub, and Maguire 2012). Thus, the spatial element of the association seems crucial to hippocampal function. It has also been suggested that anterior hippocampus flexibly re-combines episodic memories to form novel scenes (Addis et al. 2009; Martin et al. 2011; Schacter et al. 2012). We have demonstrated that the function of anterior hippocampus goes beyond this—it is engaged by perceiving scenes never experienced before by the participants, as well as by constructing scenes with the instruction to make them as unlike previous experiences as possible.

We propose that the hippocampus draws together semantic information, objects, and spatial information into a single scene representation, in agreement with frameworks such as the scene construction theory (Hassabis and Maguire 2007, 2009; Maguire and Mullally 2013) and the emergent memory account (Graham et al. 2010). Visual perception and imagination are both able to engage anterior medial hippocampus to form a representation or model of the scene. This may be supported by posterior hippocampus, but this is not required when the scene is (re)constructed endogenously based on memory or imagination (Hassabis, Kumaran, and Maguire 2007).

If anterior medial hippocampus operates to form a representation of a scene, then it should be maximally engaged by perceiving or imagining novel scenes rather than recapitulating recently constructed scenes. A number of studies have shown that anterior hippocampus responds more strongly to novel than repeated scenes. For instance, Poppenk et al. (2010) found anterior hippocampus to respond more strongly to novel relative to familiar subsequently remembered scenes. Scene novelty was also investigated by Howard et al. (2011), who manipulated the relative placement of objects, backgrounds and whole scenes presented visually. They found left anterior hippocampus was maximally activated when changing the position of an object with respect to its background, thereby altering the spatial configuration of the scene. Thus, anterior hippocampus may be sensitive to the appearance of an entirely novel scene or to a novel configuration of an existing scene. As mentioned above, we investigated novelty effects in a supplementary analysis specifically for the perceive scenes condition, and found anterior hippocampus to be engaged more by perceiving novel than repeated stimuli. Furthermore, when our participants constructed novel scenes anterior hippocampus was engaged, but not when they maintained previously constructed scenes in working memory. Novelty per se is not sufficient to explain our fMRI results, as our scene contrasts were each relative to matched baselines which used novel objects. However, a novelty effect particularly relating to scenes rather than objects would accord with our results—but is such an explanation sufficient, and what function could scene novelty serve?

One possibility is that anterior hippocampus responds to scene novelty because it is involved in incidental encoding in LTM. Indeed, anterior hippocampus was specifically implicated by Schacter and Wagner (1999) who proposed that it encodes novel associations or relations between stimuli, regardless of what the stimuli contain. Following this, several studies have implicated anterior hippocampus in encoding word–pair, picture–pair, or name–face associations (Pihlajamaki et al. 2003; Sperling et al. 2003; Prince et al. 2005; Chua et al. 2007). In a recent study, Gaesser et al. (2013) investigated hippocampal involvement in assembling the elements of imagined future events, while controlling for novelty and encoding using a modified recombination paradigm (Addis et al. 2009). When controlling for novelty and encoding, a contrast of imagining new events > re-imagining previously imagined events revealed left posterior hippocampus. Without these controls, the authors additionally found right posterior and anterior hippocampus. They concluded that construction of the event occurs in posterior hippocampus—whereas anterior hippocampus reflects only an encoding or novelty confound. The difficulty in interpreting that result stems from the re-imagine condition, which introduces an additional process of event/scene recall. If anterior hippocampus were involved in constructing novel imagined scenes in addition to constructing scenes recalled from LTM, then a difference in activation between these conditions would not be expected in anterior hippocampus. Evidence for this comes from Hassabis, Kumaran, and Maguire (2007), who performed the similar contrast of constructing novel scenarios > recalling previously constructed events (albeit without control for novelty and from a week earlier rather than a day earlier). They found scene construction and recall separately engaged anterior or mid hippocampus (c.f. Tables 4 and 5 in Hassabis, Kumaran, and Maguire 2007), with no difference in hippocampal activation between conditions.

We suggest that LTM encoding of arbitrary associations, relations or events is not a good model for explaining anterior hippocampal activation in scene perception and scene construction. First, as mentioned above, in an additional analysis of our data, we found no correlation between hippocampal activation and subsequent memory. The scene discrimination literature has also demonstrated successful performance independent of subsequent memory—Lee et al. (2013) found a main effect in bilateral anterior hippocampus for correct versus incorrect scene oddity judgments, regardless of whether the scenes were subsequently remembered or forgotten. Martin et al. (2011) had participants construct episodes (as opposed to static scenes used in this study) and report the level of detail in each, followed by a surprise memory test. They did find subsequent memory effects in anterior and posterior hippocampus, but they additionally found each of these regions to be modulated by the level of detail in the imagined scenes. From this result, they suggested that “the contribution of these hippocampal regions to encoding success might depend, at least in part, on the ability to construct a detailed and therefore memorable simulation” (p. 13861). In agreement with this, we suggest that while encoding a memory of a scene likely depends on anterior hippocampus, in order to have a scene to remember in the first place, this region must have successfully constructed it. Perceiving or imagining novel scenes places greater demand on anterior hippocampus than perceiving or re-imagining recently constructed scenes, because of the need to form a novel representation.

In summary, we propose that the activation of anterior medial hippocampus by constructing and perceiving scenes is best explained not by LTM encoding, novelty or recall, but by the process of scene construction. This is the creation of a spatially coherent representation of the scene, whether perceived or imagined. Activity in posterior hippocampus supports this process, specifically when the scene is presented visually.

### Scene Maintenance Does Not Explain Hippocampal Involvement in Scene Construction

The second question we set out to address was what aspect of scene processing is undertaken by the hippocampus—is it the construction of the scene representation, as scene construction theory proposes (Maguire and Mullally 2013), or would just maintaining a representation of a scene in working memory be sufficient to engage the hippocampus? We found no significant activation in the hippocampus for maintaining constructed scenes in working memory above and beyond that for maintaining objects, and peak activity was significantly less for maintaining constructed scenes than for constructing scenes.

One explanation for reduced hippocampal activation for maintaining scenes is that it may not have been an engaging task. However, we found that maintaining scenes was not a passive process. Two thirds of subjects reported active strategies while maintaining scenes, and even those who reported more passivity did not rate scene maintenance as any easier than constructing scenes. The fMRI results were not any different when only those 16 subjects who reported active maintenance processes were considered. We infer from these results that scene maintenance is not sufficient to explain the activity we see for scene construction. Furthermore, this also argues against the possibility that working memory load could explain the activation we observed for scene construction—both construction and maintenance required processing of the same scene online, yet activation was significantly greater for construction.

It is important to note that this study was not designed to experimentally manipulate working memory load and, thus, our results make no claims as to the involvement of the hippocampus in working memory per se. There is evidence from previous studies of working memory using scene stimuli that load may have an effect on the hippocampus, and there is indirect evidence that it may interact with scene construction. Stern et al. (2001) found hippocampal activation increased with working memory load specifically for novel scenes, but not repeated scenes, suggesting that it has an effect during the creation of the scene representation. Lee and Rudebeck (2010) investigated the interaction between working memory load and stimulus type (scenes or shapes) in posterior hippocampus and PHC, using 1-back and 2-back memory tasks. They found working memory load only had an effect for scenes and not shapes, suggesting that working memory load might have an effect in the hippocampus but only when scene representations are constructed. In a neuropsychological study, Hartley et al. (2007) investigated scene perception (a match to sample task) and working memory (a delayed match to sample task) in 4 patients with focal hippocampal lesions. Two patients had deficits in perception, but all 4 had deficits in working memory, for as little as 2-s delay. The authors inferred that scene perception may be supported by regions outside the hippocampus, whereas scene memory, even over short periods, always requires the hippocampus. In contrast, setting out to demonstrate that the hippocampus would only be engaged when working memory limits were exceeded, Jeneson et al. (2011) found that patients with hippocampal lesions were impaired at matching scenes in a demanding continuous recognition task but, in a standard study-test task, they were only impaired at longer delays. It is also important to consider in interpreting studies such as this, where the sample and test scenes differ in the arrangement of their contents, that patients' lack of a hippocampal scene model could impair performance independently of working memory demands.

In summary, while working memory load may modulate the level of hippocampal activity for maintained scenes, we found neither the maintenance of rich, naturalistic visually presented scenes nor the maintenance of endogenously constructed scenes to be sufficient to explain activity for scene perception and scene construction.

### Functional Connectivity of the Hippocampus During Scene Construction

Our third question concerned the functional connectivity of the hippocampus during scene construction. Having identified a region of hippocampus common to scene construction and scene perception, this also gave us the opportunity to explore whether this region communicates with different brain regions depending on the task, despite being engaged by both. We found significantly stronger connectivity with a widely distributed set of brain areas during scene construction compared with scene perception. The majority of the connectivity findings arose when using the right hippocampus as the seed region, with fewer results involving increased connectivity with the left hippocampus. Several previous studies involving scene construction have noted the preferential engagement of the right hippocampus (Weiler et al. 2010; Martin et al. 2011; Mullally, Hassabis, and Maguire 2012). We now extend this by showing that connectivity particularly with right anterior medial hippocampus was enhanced during scene construction. The right hippocampal involvement may be related to the visuospatial nature of the stimuli; such a preference for the right hippocampus has long been recognized (Milner 1975).

We hypothesized enhanced connectivity between the hippocampus and cortex during scene construction, reflecting the need for the hippocampus to access cortically stored scene elements to facilitate construction. In this regard, it is interesting to note the locations with which the anterior medial hippocampus showed enhanced connectivity. Left hippocampus had enhanced connectivity with left superior temporal sulcus and right hippocampus had enhanced connectivity with bilateral superior temporal sulci. Results from studies on patients with semantic dementia or with lesions to the anterior temporal lobe have implicated this part of the brain in semantic processing (Binder et al. 2009), especially in extracting meaning from words (Price 2010), with the strongest effects found when subjects are probed with more difficult semantic association tasks (Lambon Ralph et al. 2012). Similarly, studies using transcranial magnetic stimulation with healthy participants have shown that temporary inhibition of anterior temporal lobe alone causes impairments in picture naming, object naming, and synonym finding (Pobric et al. 2007, 2010), tasks intended to test semantic processing. One study which had better anatomical specificity than most (Binney et al. 2010) narrowed down this “semantic hub” specifically to anterior superior temporal sulcus as well as anterior fusiform gyrus and anterior inferior temporal gyrus, regions of high-level association cortex which may provide semantic association processing, permitting selection of the most relevant elements for inclusion into a scene.

We also hypothesized enhanced coupling between hippocampus and dorsal or ventral visual regions, associated with vivid imagining of the scenes. Both left and right anterior hippocampal ROIs had stronger connectivity with left IPL during scene construction relative to perception. Right hippocampus additionally connected with right IPL. Kravitz et al. (2011) sought to update the notion of dorsal and ventral streams for visual processing by reviewing tract-tracing studies in monkeys. Their model identified a “parieto-medial temporal” pathway whereby visual information passes between IPL and hippocampus, both directly and indirectly via RSC and PHC. They suggest a role for this pathway in directing spatial attention or translating egocentric and allocentric reference frames. Earlier in the visual hierarchy, we found PPI results in primary visual cortex for constructing more than perceiving scenes using right anterior hippocampus as the seed region. Greater connectivity between right hippocampus and early occipital areas could reflect the hippocampus accessing reactivated sensory representations, in keeping with previous studies showing that vivid mental imagery engages occipital cortex (Ganis et al. 2004; Chadwick et al. 2013) and that activity in early visual areas correlates with the vividness of mental imagery (Cui et al. 2007). Indeed, a key feature of scene construction is vividly experiencing a spatially coherent scene in the mind's eye, and it is this experience which is lost by patients with hippocampal lesions (Mullally, Intraub, and Maguire 2012). Our results suggest that a disconnection between hippocampus and early visual areas, a pathway thought to be mediated by PHC and RSC (Byrne et al. 2007), could play a role in the patients' scene construction deficit.

In a supplementary analysis (Supplementary Text 5) we tested for connectivity between posterior hippocampus, which was only engaged by the perceive scenes condition relative to object baselines, and the rest of cortex. We did not find significant connectivity with early visual cortex as we would have predicted, but rather specific coupling with PHC when subjects visually perceived scenes. Further PPI analyses involving posterior collateral sulcus, a region of the ventral visual stream on the occipital/temporal cortex border, showed coupling with both PHC and early visual cortex. A dissociation may be made between our findings in anterior and posterior hippocampus. Anterior hippocampus had stronger connectivity with early visual cortex during scene construction than perception, potentially because anterior hippocampus drives production of sensory imagery. Posterior hippocampus was only engaged during scene perception, and it communicated with PHC, through which it may have received visual information via the ventral visual stream.

Finally, we also found greater PPI connectivity between bilateral anterior hippocampi and multiple loci in dorsolateral prefrontal cortex for scene construction more than perception. This region is anatomically connected to the hippocampus via presubiculum, PHC, and RSC (Aggleton 2012), and is reliably engaged as part of the “core network” for episodic memory and scene construction (Spreng et al. 2009; Summerfield et al. 2010). It has been implicated in attention and monitoring (Markett et al. 2014), raising the possibility that these frontal regions may be involved in executive control within the scene construction network.

It has previously been suggested that the hippocampus acts a hub of connectivity in memory formation and consolidation (Battaglia et al. 2011) and was identified as one of a “rich club” of 12 highly connected brains hubs (van den Heuvel and Sporns 2011). Our PPI results indicate which regions it may communicate with during scene construction. Based on our PPI analyses, we hypothesize that successful communication between anterior medial hippocampus and areas within parietal, frontal and temporal cortices underpins scene construction, which may explain why hippocampal lesions are so detrimental to scene construction, and to other functions such as episodic memory and spatial navigation that also rely on hippocampal interaction with multiple brain regions.

### Scene Construction and Scene Perception: a Synthesis

Scene construction, scene perception, and working memory maintenance for scenes were addressed as 3 separate conditions in this study; however as already mentioned, they are unlikely to be independent. Scene construction and scene perception share common activation in anterior medial hippocampus, and it is likely that both depend on processes of working memory maintenance. As mentioned in the Introduction, there is evidence on both sides of the debate as to whether the hippocampus is involved in perception. We suggest that a model based on scene construction can help elucidate these disparate results.

When constructing a scene, a representation is created in the hippocampus. Several processes may feed into this. LTM recall contributes elements to be added into a constructed scene, as does information from visual perception, if available. Encoding and maintaining the representation in working memory enables the scene to be constructed over an extended period of time, without loss of detail while the scene is being constructed. If and only if scene construction has occurred, dependent processes are facilitated. For instance, discrimination tasks where scenes are shown from different viewpoints can only be completed successfully if a coherent spatial model has been constructed. Similarly, LTM encoding depends on the scene representation having been constructed, in order for it to be consolidated into LTM. BE only occurs if a scene model has first been encoded. We propose this model is instantiated in a distributed scene network, including anterior medial hippocampus which mediates the distributed scene representation and posterior hippocampus and PHC which integrate the visually perceived scene into the representation. Of course, the interaction between scene construction and dependent processes is likely to be more complex than described. The hippocampal scene model, for instance, could reciprocally provide top-down expectations or priors onto early visual cortex (Chadwick et al. 2013). Similarly, pattern completion in the hippocampus would drive additional retrieval from LTM (Marr 1971).

Under this account, differences in the wider scene perception literature may be explained by whether participants needed to construct spatially coherent scenes. This is in agreement with other authors who have suggested that the hippocampus is involved in scene perception when an allocentric, viewpoint-independent representation is employed for the task (Hartley et al. 2007; Lee et al. 2012; Aly et al. 2013). Scene construction in the perceptual domain may accord with strength-based perception of Aly et al. (2013), but it goes further by placing scenes at the center of hippocampal function and proposing a common process across perception, imagination and episodic memory (Maguire and Mullally 2013). In this study, we have provided evidence that this common construction process is supported by anterior medial hippocampus. Importantly, while scene perception can engage the hippocampus, the hippocampus is not required for perceiving scenes. Patients with hippocampal lesions tested by Mullally, Intraub, and Maguire (2012) and Race et al. (2013) could richly describe scenes that were put in front of them, which in the latter study included forming detailed narrative descriptions of scene images. If so much can be achieved without the hippocampal scene representation, what purpose might it serve? Mullally, Intraub, and Maguire (2012) found that when these same patients were asked to imagine stepping back from the photographs, they could list the objects they might see, but they could not describe the spatial relationships which would exist between them. In a similar vein, when we perceive scenes, we are subject to the scene-specific cognitive phenomenon of BE. The BE effect depends on scene construction, as the world beyond the scene borders must be internally generated. Thus, it may be that healthy individuals are never passively perceiving scenes because the BE effect, underpinned by scene construction, always occurs and engages the hippocampus. Thus, without a hippocampal model being constructed, the scene currently in view can only be comprehended in isolation and cannot be extended beyond its borders or in one's imagination.

### Summary and Conclusions

In this study, we found that a common region of anterior medial hippocampus is engaged by viewing scenes and constructing scenes in the imagination. We showed that this activation cannot be explained by maintenance of the scene representation alone, providing additional evidence that hippocampus is involved in the construction of the scene representation. We also explored the functional connectivity of this region for perception and construction of scenes, and demonstrated a wide network of connections for scene construction. Our results support the notion that scenes are important for understanding the role of the hippocampus, and that scene construction may extend beyond memory and imagination into online scene perception.

## Supplementary material

Supplementary material can be found at http://www.cercor.oxfordjournals.org/.

## Funding

This research was funded by the Wellcome Trust (E.A.M.) and the Brain Research Trust (P.Z.). Funding to pay the Open Access publication charges for this article was provided by The Wellcome Trust.

## Supplementary Material

Supplementary Data
