# Supplementary material for: Constructing, Perceiving, and Maintaining Scenes: Hippocampal Activity and Connectivity
Source: Cereb Cortex. 2014 Nov 18;25(10):3836–55. doi: 10.1093/cercor/bhu266 (PMC4585517; doi:10.1093/cercor/bhu266)
Supplement: Supplementary Data [file supp_25_10_3836__index.html]

Constructing, Perceiving, and Maintaining Scenes: Hippocampal Activity and Connectivity — Supplementary Data 

# Constructing, Perceiving, and Maintaining Scenes: Hippocampal Activity and Connectivity

## Supplementary Data

Supplementary Data

**Files in this Data Supplement:**

- Supplementary Data - Docx file
