# Supplementary material for: Constructing, Perceiving, and Maintaining Scenes: Hippocampal Activity and Connectivity
Source: Cereb Cortex. 2014 Nov 18;25(10):3836–55. doi: 10.1093/cercor/bhu266 (PMC4585517; doi:10.1093/cercor/bhu266)

**Supplementary Material**

**Constructing, perceiving and maintaining scenes: Hippocampal activity and connectivity**

Peter Zeidman, Sinéad L. Mullally, and Eleanor A. Maguire

**Text S1**

Prior to scanning, participants were trained on the task requirements. The instructions they were given are reproduced below. These instructions were re-iterated in an abridged form at the end of the training, and again over the intercom once the participant was lying in the scanner. The need for creativity and novelty in the construct conditions was particularly emphasised. To test whether participants understood what was required of them in the construct conditions during training, participants constructed scenes and objects out loud, without the time constraints of the real scanning experiment. The experimenter then fed back whether any instructions were not followed. If any participant had constructed scenes or objects which clearly referred to a specific experience, for instance through use of the past tense, then it would have been fed back to them that this was an error. This was not necessary for any participant. We then further tested compliance with the instructions in the post-scan debrief questions, described in the main text.

*Perceive scenes*:

“Sometimes, you’ll see the words “View Scene” on the screen. After this you’ll see a picture of a scene, something like this <Demo Scene 1>. Your task is simply to look at the scene, as if it were really in front of you. Importantly, don’t try to imagine what’s beyond the edges of the picture and don’t imagine yourself moving round the scene. Also, don’t think of memories of any scenes like this from your past. Instead, just focus on looking at the scene, as if it were really in front of you. Don’t think about anything else, just focus on looking at the scene.”

*Perceive objects*:

“At other times in the experiment, you’ll see the words “View Object”. This will then be replaced with a picture of an object, something like this <Demo Object 1>. Like before, just look at the object as if it were in front of you. But importantly, try not to think of things associated with it or similar things you have seen before. Just try to focus on the object in front of you. Don’t think about anything else, just focus on looking at the object.”

*Construct scenes*:

“As soon the screen goes blank, you should start imagining the scene as if it were really in front of you. In the time you are given, try to make the image really clear and vivid. The scene you create in your imagination should be new and original. It shouldn’t be a memory from your past, or something you’ve seen in a movie. Like when you viewed the photo of a scene, keep your mental “camera” in a fixed position when you’re imagining - don’t imagine moving around the scene, don’t zoom in or change your viewpoint. And as I’ve said, don’t think about memories of scenes from your past. We’d like you to construct something new.”

*Construct objects*:

“The two words on the screen may describe an object. If so, you should imagine this single object as if it were in front of you. And, just like the photo you saw, you should imagine the object on its own against a plain white background. Try not to think of an actual object you’ve seen before - again we’d like you to come up with something new. Don’t think about other objects you’d associate with it, or locations you might find it. Don’t spin the object round. Just imagine it on its own, against a plain background, perfectly still. If you find you’re doing this quickly, just keep mentally ‘looking’ at and focussing on the object until the time is up.”

*Maintain scenes*:

“So I’ve shown you viewing a scene or an object, and imagining a scene or an object. A third kind of thing that might happen, after you’ve just viewed or imagined a scene, is that you’ll see the words <Maintain Scene>. This tells you that when the screen next goes blank, your task is to maintain, in your mind’s eye, the scene that you just viewed or imagined. So let me show you an example of this. You see <View Scene>, so you know a picture’s coming. Then you view the scene <Demo Scene 1>, remembering not to imagine what’s beyond the edges or think about memories. Then you get the instruction <Maintain Scene>, and when the screen goes <Blank> you bring back or ‘maintain’ the scene in your mind’s eye. Please don’t add any detail or change anything. Just bring it back to mind, and hold it in your mind’s eye as if it were really in front of you and you were looking at it once again. Keep your eyes open throughout. After a few seconds, the frame will automatically disappear and the cross will come up <Fixation> to tell you to stop.”

*Maintain objects*:

“A similar thing will happen for objects. For example, you’ll see two words like <Demo Object Cue 1>. As I explained, when the screen goes <Blank> you’ll imagine that object against a plain background. Then, if you see <Maintain Object>, you bring that object back into your imagination, as if it were really in front of you and you were looking at it once again. Please don’t add anything, change anything, or move the object. Just keep everything the same. Does that make sense?”

*Vigilance task*:

“During scanning there’s one more kind of task you’ll be asked to perform. This one’s quite simple. You’ll see the instruction <Pay Attention>. This will be followed by a <Green Cross>. You don’t have to do any imagining here. Your task is simply to look at the green cross, and count the number of times it flashes red. On some trials it won’t change at all, on others it may flash red once, or twice. It can change colour at any time during the trial (which is about 15 seconds long) so you’ll need to pay attention throughout and wait until the end to make your decision. The cross will turn white when the trial is over, at that point if there was no change in colour do nothing, if it changed colour once, press this key <show them>, and if it changed colour twice press this key <show them>.”

**Text S2**

Our behavioural analysis demonstrated that four participants may have engaged in processes beyond passive scene viewing, such as imagination. It was therefore important to confirm that these subjects were not driving our finding of hippocampal activity for perceiving scenes in anterior and posterior hippocampus. We therefore re-calculated our ROI analyses with the 21 participants who reported only passive scene viewing.

We conducted an SPM analysis within the hippocampal ROIs, using the same setup as the main text (FWE-corrected statistics with SnPM toolbox on unsmoothed data, with each condition against its matched object baseline, p < 0.05). This demonstrated voxels with significant activity throughout bilateral hippocampi for perceiving scenes and in anterior hippocampi for constructing scenes. There was one significant voxel in left posterior hippocampus for maintaining perceived scenes and none for maintaining constructed scenes, matching the findings from the main analysis. We then extracted peak activations for each condition and entered them into a repeated measures ANOVA with factors of hemisphere (left, right), region (anterior, posterior) and scene condition (perceive, construct, maintain perceived and maintain constructed), all with object baselines subtracted. There was no significant main effect of hemisphere (F(1,20)=0.547, p=0.47) nor any interaction involving hemisphere. There was a main effect of region (F(1,20)=9.67, p=0.006) and of condition (F(3,60)=17.76, p=2.3e-8) and a significant interaction between them (F(3,60)=5.43, p=0.002). This profile of results matched the analysis in the main text, and post-hoc *t*-tests reproduced the result of significantly greater activation for perception than construction in anterior (t(20)=3.53, p=0.002) and posterior (t(20)=3.17, p=0.005) hippocampus, and significantly greater activation for perceiving than maintaining perceived scenes in anterior (t(20)=4.62, p=1.65e-4) and posterior (t(20)=4.19, p=4.48e-4) hippocampus. As per the results with all subjects, there was significantly greater activation for constructing than maintaining constructed scenes in anterior (t(20)=4.14, p=5.03e-4) but not posterior (t(20)=0.69, p=0.50) hippocampus.

**Text S3**

We re-ran the ROI analysis ANOVA on only the 16 participants who reported using an active process during the maintain condition (as reported in Figure 4 in the manuscript for all participants). The ANOVA had factors of hemisphere (left, right), region (anterior, posterior) and scene condition with object baselines subtracted (perceive, construct, maintain perceived, maintain constructed). There was no significant main effect of hemisphere (F(1,15)=0.10, p=0.76), nor any interactions involving hemisphere. There was a main effect of region (F(1,15)=4.66, p=0.05) and of condition (F(3,45)=12.86, p=3e-6), as well as a region x condition interaction (F(3,45)=3.80, p=0.02). This was the same profile of responses as reported in the manuscript with all subjects. Collapsing over hemisphere, post-hoc paired-sample t-tests confirmed significantly greater activation for constructing scenes than maintaining constructed scenes in anterior hippocampus (t(15)=4.01, p=0.001) and not posterior hippocampus (t(15)=1.37, p=0.19), reflecting our finding of scene construction being limited to anterior hippocampus. There was significantly greater activation in anterior hippocampus for perceiving scenes than maintaining perceived scenes (t(15)=3.76, p=0.001) and in posterior hippocampus (t(15)=3.10, p=0.007). Thus, the reduced activation we observed for maintenance was not driven by the subjects who reported being passive during the maintain scenes condition.

**Text S4**

We investigated the effect of stimulus novelty on hippocampal involvement in perceiving scenes. If the activation for perceiving scenes could be fully explained by stimulus novelty, then we would expect no hippocampal activation for perceiving a familiar scene. To test this, we compared two conditions from our original experimental design (Figure S1, top). In condition D, participants perceived one scene and then another different scene. In condition E, participants perceived one scene and then the same scene again. All scenes were trial unique.

The conditions were modelled in a general linear model as follows. As for the analyses described in the main text, each trial was split into two parts (the first 9.5s and the second 9.5s). Regressors were included for perceiving novel scenes in the second part of condition D (“perceive novel”) and for perceiving repeated scenes in the second part of condition E (“perceive repeated”). A further regressor modelled perceiving scenes in the first half of trials, but was not used here. All other conditions (construct, maintain constructed, maintain perceived) were modelled as described in the main text. Note that the comparison of interest – perceive novel vs perceive repeated – only included the second half of conditions D and E, and thus were of reduced power compared to the results in the main text.

Perceiving novel scenes gave significant activation of voxels in both anterior and posterior hippocampi (Figure S1, bottom left). Crucially, perceiving repeated scenes also gave significant hippocampal activation, but only in the posterior hippocampus (Figure S1, bottom right). Peak responses for each condition were extracted (Figure S2) and analysed using a repeated measures ANOVA with factors of hemisphere (left, right), region (anterior, posterior) and novelty (novel, repeated). There was a significant main effect of hemisphere (F(1,24)=6.77, p=0.016), region (F(1,24)=5.27, p=0.03) and novelty (F(1,24)=12.73, p=0.002). No two- or three-way interactions were significant. Post-hoc paired-sample *t*-tests showed significantly greater response for novel than repeated stimuli in left anterior (t(24)=2.64, p=0.01), right anterior (t(24)=3.51, p=0.002) and right posterior hippocampus (t(24)=2.66, p=0.01), but not left posterior hippocampus (t(24)=2.05, p=0.05).

Together, these results demonstrate that novelty enhances the response of the hippocampus to visually perceived scenes. There was a stronger response in anterior hippocampus to scenes than objects when the stimuli were novel. By contrast, posterior hippocampus responded more strongly to scenes than objects regardless of novelty. This pattern of responses fits with our proposition that anterior hippocampus performs scene construction, placing it under greatest demand when a scene is novel, whereas posterior hippocampus has a role in ongoing visual scene perception even in the absence of novelty.

**Text S5**

Having found that posterior hippocampus had greater activation for perceiving scenes than objects (a pattern not observed for constructing or maintaining scenes), we investigated the connectivity of posterior hippocampus when perceiving scenes. Seed regions for left and right posterior hippocampus were defined as all significant voxels for the contrast of Perceive Scenes – Perceive Objects in the hippocampus (see Figure 3 in the main text), posterior to coordinate y=-21, the border of the uncus. There was no overlap with the anterior medial hippocampus mask used in the main analysis. All other experimental methods matched those of the PPI analysis in the main text.

Using the seed region of right posterior hippocampus, there was significantly stronger connectivity for perceiving scenes than objects only with left PHC (-33,-39,-12; z=3.91). Conversely, using the seed region of left posterior hippocampus, there was there was significantly stronger connectivity only with right PHC (33,-39,-9; z=3.77). No other activations resulted from this analysis.

We were interested to test how information from occipital cortex reached PHC / posterior hippocampus during scene perception. We revisited the Perceive Scenes – Perceive Objects contrast, and identified two regions in occipital cortex to use as PPI seeds. (These were sub-peaks of the first cluster reported in Table S1.) One was in lingual gyrus, ventral to the calcarine sulcus (left: -3,-78,-3; z>7.79, right: 6,-78,-3; z>7.79). The other was a more inferior and lateral occipto-temporal region, centred on the collateral sulcus (left: -24,-75,-12; z>7.79; right: 27,-72,-9; z=7.54), posterior to the PHC (also known as ‘PPA’). Seed regions were defined as spheres of radius 4mm positioned at these peaks.

We found no significant PPI connectivity for the contrast of Perceive Scenes – Perceive Objects with the seed in left lingual gyrus. Placing the seed in right lingual gyrus gave bilateral dorsal thalamus (left: -18,-9,27; z=4.01, right: 21,-21,30; z=3.81). With the seed region in left posterior collateral sulcus, we found significant PPI connectivity with RSC (left: -18,-60,15; z=5.82, right: 18,-54,12; z=5.69), PHC (left: -24,-45,-9; z=5.10, right: 24,-39,-12; z=5.34) and primary visual cortex in the right calcarine sulcus (15, -72, 9; z=3.62) and left lingual gyrus (-9,-78,-6; z=3.60). We found similar results with the right posterior collateral sulcus as the seed, with activations in RSC (left: -18,-59,9; z=4.51, right: 12,-51,9; z=5.36), PHC (left: -24,-51,-91; z=5.18, right: 27, -45, -6; z=5.10) and left lingual gyrus (-9,-72,-3; z=3.91).

To summarise these PPI analyses, we found that posterior hippocampus had stronger connectivity with PHC when perceiving scenes than when perceiving objects. This finding was more restricted than the connectivity of anterior medial hippocampus when perceiving scenes, which additionally had connections with occipito-parietal junction, RSC and cerebellum (see main text, Table 2). When we placed PPI seeds in posterior collateral sulcus, we found this region connected with both early visual areas and regions known to be particularly responsive to scenes (RSC and PHC). Based on these results and established anatomical studies of dorsal and ventral visual streams (for review see Kravitz et al. 2011), we suggest that posterior hippocampus interacts with early visual areas via PHC during scene perception. Note that we did not examine the Construct Scenes or Maintain Scenes conditions here, as posterior hippocampus was only significantly engaged by our Perceive Scenes condition, and our regions were selected on the basis of the Perceive Scenes – Perceive Objects contrast.

Kravitz, DJ, Kadharbatcha SS, Baker CI, Mishkin M. 2011. A new neural framework for visuospatial processing. Nat Rev Neurosci 12(4):217-230.

**Table S1** Whole-brain contrasts for each condition relative to its object baseline condition.

| **Perceive Scenes – Perceive Objects** | | |
| --- | --- | --- |
| **XYZ** | **Z** | **Region** |
| -27,-51,-9 | > 7.14 | L Collateral sulcus  Bilateral lingual gyrus |
| 3,-54,-36 | 7.14 | R Cerebellum  L Cerebellum |
| -21,-30,-3 | 6.92 | L Posterior thalamus  L Anterior medial hippocampus |
| 21,-12,-21 | 6.34 | R Anterior medial hippocampus |
| 51,-6,-21 | 5.88 | R Superior temporal sulcus |
| 3,48,-18 | 5.71 | R vmPFC |
| -6,-78,-39 | 5.67 | L Cerebellum |
| -42,-75,18 | 5.08 | L Occipital gyrus |
| 39,-45,-24 | 5.06 | R Fusiform gyrus |
| -6,45,-15 | 5.01 | vmPFC |
| **Construct Scenes – Construct Objects** | | |
| **XYZ** | **Z** | **Region** |
| -24,-42,-12 | 6.79 | L PHC |
| -21,-18,-21 | 6.78 | L Anterior medial hippocampus |
| 24,-30,-21 | 6.58 | R PHC  R Anterior medial hippocampus |
| 6,-42,6 | 6.57 | R RSC  L Retrosplenial cortex |
| -39,-72,30 | 6.26 | L Posterior parietal cortex |
| 33,-78,36 | 5.85 | R Posterior parietal cortex |
| 3,-60,54 | 5.79 | R Precuneus |
| -60,-6,-18 | 5.19 | L Superior temporal sulcus |
| -6,-51,-45 | 5.14 | L Cerebellum |
| 9,-51,-42 | 4.93 | R Cerebellum |
| -9,36,-12 | 4.91 | L vmPFC |
| **Maintain Perceived Scenes – Maintain Perceived Objects** | | |
| **XYZ** | **Z** | **Region** |
| 3,-78,39 | 6.22 | R OPJ  Precuneus |
| -18,-60,18 | 5.93 | L OPJ |
| 30,-36,-15 | 5.58 | R PHC |
| 9,-42,0 | 5.45 | R RSC |
| -21,-36,-18 | 5.32 | L PHC |
| -15,-54,6 | 5.27 | L RSC |
| -6,45,3 | 5.26 | L RSC |
| 21,-60,21 | 5.25 | R OPJ |
| -36,-81,30 | 5.18 | L Posterior parietal cortex |
| 33,-81,36 | 4.99 | R Posterior parietal cortex |
| 33,-81,27 | 4.99 | R Posterior parietal cortex |
| 9,-90,24 | 4.91 | R Dorsal occipital cortex |
| -3,-51,57 | 4.85 | L Precuneus |
| **Maintain Constructed** **Scenes – Maintain Constructed Objects** | | |
| **XYZ** | **Z** | **Region** |
| 15,-54,18 | 5.16 | R OPJ |
| -12,-54,6 | 5.12 | L OPJ |
| 9,-54,6 | 5.12 | R OPJ |
| 21,-39,-15 | 5.11 | R PHC |

Results shown at p < 0.05 FWE-corrected, minimum extent 2 voxels. OPJ = Occipito-parietal junction, RSC = Retrosplenial cortex, PHC = parahippocampal cortex, vmPFC = ventromedial prefrontal cortex

**Table S2** Whole-brain contrasts comparing conditions with their object baselines subtracted (the interaction between condition and stimulus type).

| **(Construct Scenes – Objects) - (Perceive Scenes – Objects)** | | |
| --- | --- | --- |
| **XYZ** | **Z** | **Region** |
| 9,-87,-30 | 5.48 | R Cerebellum |
| -39,-72,36 | 5.29 | L Posterior parietal cortex |
| 36,-84,-36 | 5.14 | R Cerebellum |
| **(Perceive Scenes – Objects) - (Construct Scenes – Objects)** | | |
| **XYZ** | **Z** | **Region** |
| -3,-78,-3 | > 6.76 | L Calcarine sulcus  L Lingual gyrus, bilateral occipital cortex |
| 21,-27,8 | 6.76 | R Posterior thalamus |
| -21,-30,3 | 6.16 | L Posterior thalamus |
| 0,-51,-36 | 5.65 | Cerebellum |
| 18,-42,-45 | 5.46 | R Cerebellum |
| -6,-75,-39 | 5.44 | L Cerebellum |
| -15,-72,30 | 5.40 | L OPJ |
| -42,-75,15 | 5.22 | L Posterior parietal cortex |
| **(Construct Scenes – Objects) – (Maintain Constructed Scenes – Objects)** | | |
| *None significant at p < 0.05 FWE-corrected for the whole brain* | | |
| **(Perceive Scenes – Objects) – (Maintain Perceived Scenes – Objects)** | | |
| **XYZ** | **Z** | **Region** |
| -9, -78, -9 | > 6.94 | L Inferior occipital cortex  Primary occipital cortex |
| -21,-27,-3 | 6.94 | L Posterior thalamus |
| -21,-39,-45 | 5.81 | L Cerebellum |
| 24,-27,0 | 5.79 | R Posterior thalamus |
| -18,-72,21 | 5.29 | L OPJ |
| 15,-84,36 | 5.02 | R Dorsal occipital cortex |
| **(Maintain Constructed Scenes – Objects) - (Maintain Perceived Scenes – Objects)** | | |
| *None significant at p < 0.05 FWE-corrected for the whole brain* | | |
| **(Maintain Perceived Scenes – Objects) - (Maintain Constructed Scenes – Objects)** | | |
| *None significant at p < 0.05 FWE-corrected for the whole brain* | | |

Results shown at p < 0.05 FWE-corrected, minimum extent 2 voxels. OPJ = Occipito-parietal junction.

**Table S3** Correlations between peak SPM parameter estimates in the hippocampus and subsequent memory performance.

| **Region** | **Condition** | **R** | **P (uncorrected)** |
| --- | --- | --- | --- |
| Left anterior | Perceive scenes | 0.35 | 0.09 |
|  | Construct scenes | 0.31 | 0.13 |
|  | Perceive objects | 0.39 | 0.05 |
|  | Construct objects | 0.08 | 0.69 |
| Right anterior | Perceive scenes | 0.08 | 0.70 |
|  | Construct scenes | 0.01 | 0.96 |
|  | Perceive objects | 0.06 | 0.77 |
|  | Construct objects | 0.21 | 0.32 |
| Left posterior | Perceive scenes | 0.30 | 0.14 |
|  | Construct scenes | 0.32 | 0.12 |
|  | Perceive objects | 0.15 | 0.48 |
|  | Construct objects | 0.13 | 0.53 |
| Right posterior | Perceive scenes | -0.29 | 0.16 |
|  | Construct scenes | 0.23 | 0.26 |
|  | Perceive objects | 0.15 | 0.49 |
|  | Construct objects | 0.35 | 0.09 |

No correlations were significant at the Bonferonni corrected-threshold for four correlation tests per region of p<0.0125, nor at the uncorrected threshold of p < 0.05.

**Supplementary Figure Captions**

**Figure S1** Comparison of hippocampal responses to novel and repeated scenes and objects. **Top**: The two trial types from the main analysis used in this supplementary analysis. In condition D (top row), participants viewed a scene and then a novel scene. In condition E (bottom row), participants viewed a scene and then viewed the same scene. The highlighted section of the trials in conditions D and E were compared for this analysis. **Bottom Left**: Activations for perceiving novel scenes relative to perceiving novel objects. **Bottom Right:** Activations for perceiving repeated scenes relative to perceiving repeated objects. Activations shown in 3D projection on group-average hippocampi, at p < 0.05 corrected for the small volume of bilateral hippocampi. See Text S4 for details.


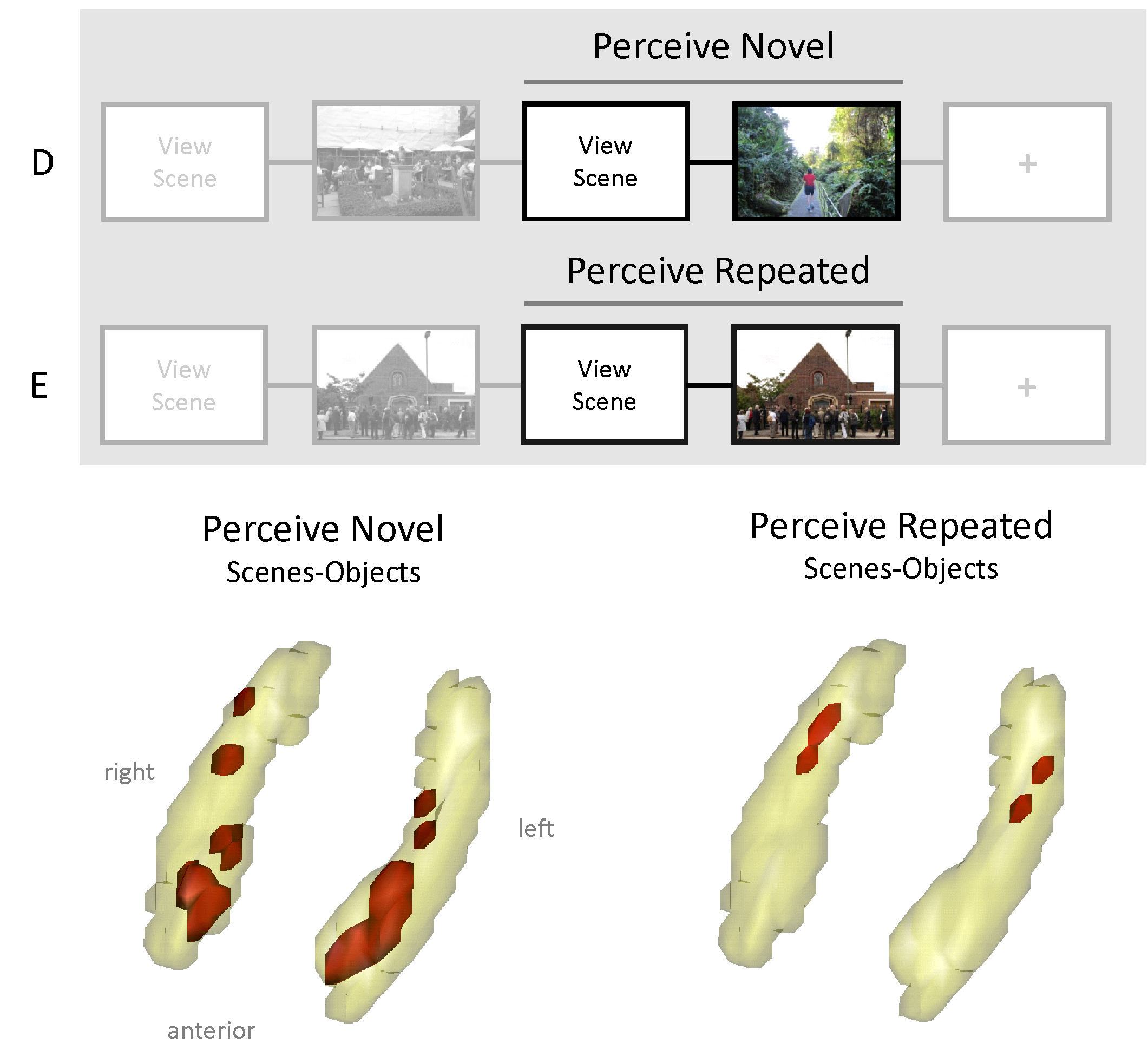


**Figure S2** Plots of hippocampal responses to novel and repeated scenes and objects.

The four plots detail group-average ROI results from left anterior, right anterior, left posterior and right posterior hippocampi. Each plot shows the peak contrast result for perceiving novel scenes > perceiving novel objects (left bar, checked) and for perceiving repeated scenes > perceiving repeated objects (right bar, dashed). Error bars indicate standard error across subjects. * post-hoc *t*-tests at p < 0.05 (see full statistics in Text S4).


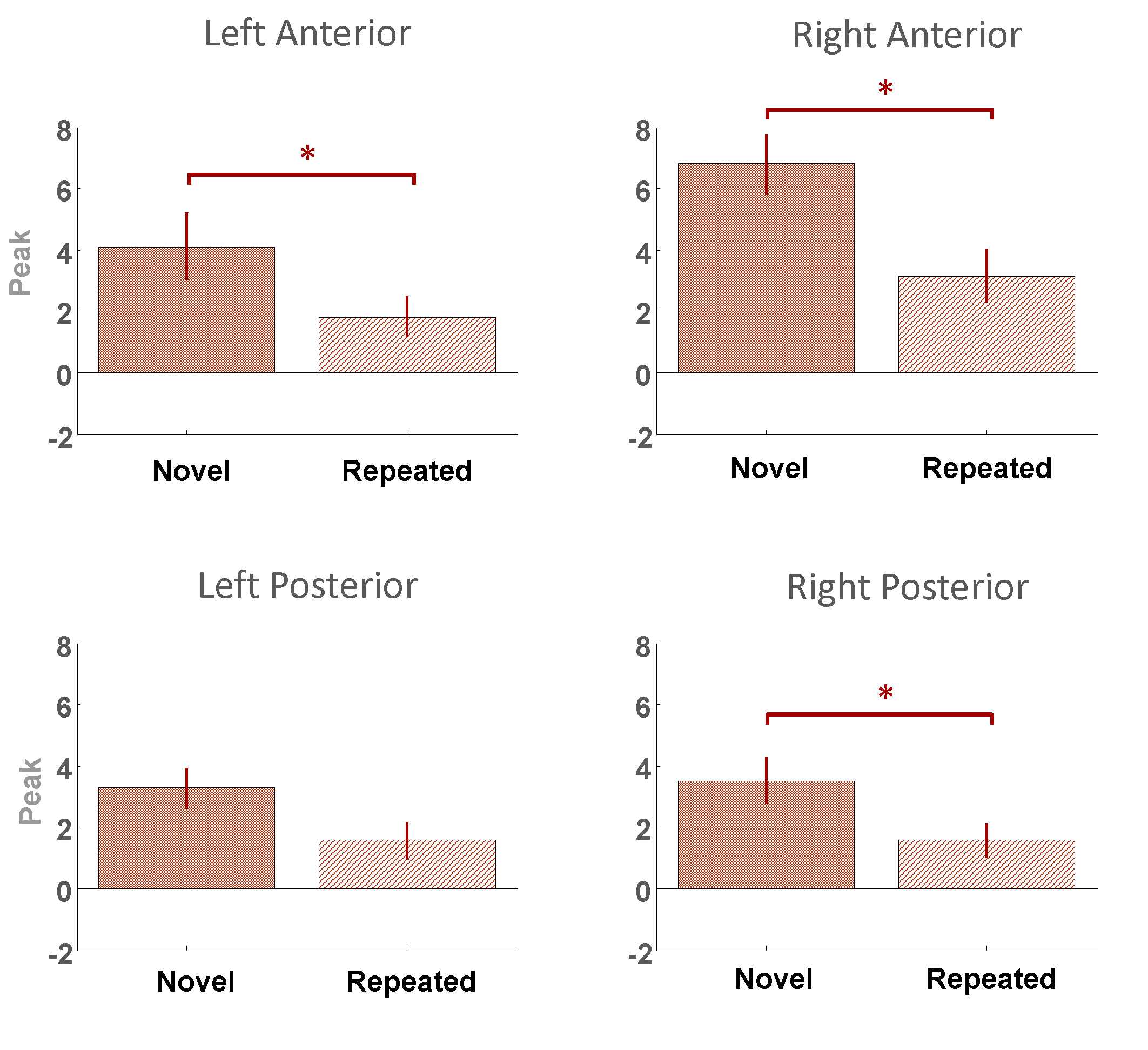

Supplement: Supplementary Data [file supp_bhu266_bhu266supp.docx]
